# Dissolvable microneedles loaded with denosumab alleviate knee osteoarthritis in rodent and canine models by inhibiting macrophage senescence

**DOI:** 10.7150/thno.116970

**Published:** 2026-01-01

**Authors:** Chaochang Ming, Duohang Bi, Hongtao Tian, Weijian Liu, Haitao Li, Yuxiang Hu, Zhenyu Song, Dongdong Xu, Hao Xu, Hongyan Li, Shenghui Lan, Weihua Xu, Wei Chen, Qiong Li, Jiawei Feng, Qianqian Cao, Xiaoyang Wang, Panlong Fan, Jintao Zhu, Wei Yu, Chaoge Ming, Zhipeng Dai, Yijing Liu, Wei Tong

**Affiliations:** 1 Department of Orthopedics, Union Hospital, Tongji Medical College, Huazhong University of Science and Technology, Wuhan, 430022, Hubei, China.; 2 Department of Orthopedics, Henan Provincial People's Hospital, People's Hospital of Zhengzhou University, Zhengzhou 450003, Henan, China.; 3 Department of Orthopedics, Xinxiang Medical University, Xinxiang 453003, Henan, China.; 4 Hubei Key Laboratory of Bioinorganic Chemistry and Materia Medica, Hubei Research Center for Biomaterials and Medical Protective Materials, School of Chemistry and Chemical Engineering, Huazhong University of Science and Technology (HUST), Wuhan 430074, Hubei, China.; 5 Department of Orthopedics, Affiliated Hospital of Guizhou Medical University, Guiyang 55000, China.; 6 The Second Affiliated Hospital of Guilin Medical University, Guilin, 541000, Guangxi, China.; 7 Department of Orthopedics, The Eighth People's Hospital, Jiangsu University, Shanghai 200235, China.; 8 Department of Orthopedics, Xuhui Branch of The Sixth People's Hospital, Shanghai Jiao Tong University, Shanghai 200233, China.; 9 Department of Orthopedics, The Third Hospital of Hebei Medical University, Shi Jiazhuang 050051, Hebei, China.; 10 Department of Radiology, First People's Hospital of Jiangxia District, Wuhan, 430022, Hubei, China.; 11 Department of Orthopedics, Huaxian Orthopedic Hospital, Anyang 456400, Henan, China.

## Abstract

**Rationale:** Osteoarthritis (OA) lacks disease-modifying therapies. Although systemic denosumab delays OA progression, it causes uneven drug distribution and off-target effects, whereas intra-articular injections are invasive and risk joint infection. We aimed to develop a minimally invasive microneedle platform that delivers denosumab locally to achieve therapeutic efficacy comparable to intra-articular injection while avoiding systemic exposure.

**Methods:** A dissolvable denosumab-loaded microneedle array (MNs@De) was fabricated for transcutaneous intra-articular delivery. OA was induced in rodents and Beagle dogs; animals were treated with MNs@De, systemic denosumab, intra-articular denosumab, or vehicle. Synovial inflammation, cartilage erosion, and pain were evaluated histologically and behaviorally. Single-cell RNA sequencing and immunofluorescence were performed to assess macrophage senescence and chondrocyte metabolism. Secretion of pro-inflammatory and catabolic factors was quantified *in vitro* using senescent macrophage-chondrocyte co-cultures.

**Results:** MNs@De delivered denosumab effectively into joints, significantly reducing synovial inflammation, cartilage erosion, and pain compared with systemic administration and achieving outcomes comparable to intra-articular injection. Single-cell profiling revealed that denosumab markedly decreased senescent macrophage abundance within synovial tissue. Mechanistically, denosumab inhibited senescent macrophage-derived pro-inflammatory and catabolic factor release, thereby shifting chondrocytes from catabolic to anabolic states.

**Conclusions:** Targeting senescent macrophages via MNs@De attenuates OA progression without requiring intra-articular injections or increasing systemic drug exposure. Microneedle-mediated denosumab delivery offers a minimally invasive, localized therapeutic strategy for OA.

## Introduction

Osteoarthritis (OA) is one of the most common degenerative joint diseases and a chronic disabling condition affecting 500 million people worldwide 1, 2. One major challenge in treating OA is the lack of disease-modifying drugs, likely due to the complex and not fully understood mechanisms of the condition. First-line medications mainly consist of non-steroidal anti-inflammatory drugs (NSAIDs), which act as analgesics 3, 4, but they come with gastrointestinal side effects and risks of cardiovascular and cerebrovascular diseases 5. Consequently, discovering and developing novel agents with robust disease-modifying potential remains a top priority in OA research.

Denosumab (De) is a monoclonal antibody that inhibits the binding of receptor activator of nuclear factor kappa-B ligand (RANKL) to its receptor, RANK, and suppresses the activation and differentiation of monocyte-macrophage lineage cells and subsequently osteoclasts. This action results in anti-inflammatory effects and reduces bone resorption, which is closely associated with OA. Recently, systemic injection of De was shown to alleviate mouse knee OA by protecting cartilage from degeneration 6, and to delay hand OA progression in patients 7. We are also currently performing a Phase II clinical trial of De on patient knee OA and found decreased synovial inflammation and pain, with increased joint function after 6 months of treatment (NCT06357741). However, OA is a localized joint disease, making local treatments, such as intra-articular injection, more favorable due to their higher drug concentration and reduced systemic side effects. Unfortunately, intra-articular injection may result in joint infection, a disaster for patients and much more severe than OA itself. In addition, the drug is cleared rapidly from the joint cavity, which necessitates repeated injections 8, 9. Moreover, it is not practical for small joints such as the hand 10. Consequently, there is a pressing need for innovative therapeutic approaches that can enhance treatment efficacy while minimizing adverse reactions by developing novel drug delivery systems 11, 12.

Transdermal delivery is recognized as an important local delivery method, offering benefits such as reduced systemic side effects and improved patient compliance 13. Moreover, the soft tissue corridor between the prepatellar skin and the joint capsule is short, consisting mainly of loose connective tissue and superficial bursae 14. Furthermore, daily flexion and extension can create pressure fluctuations of about ± 5 mmHg in the joint cavity, aiding the pulsatile delivery of drugs from interstitial fluid to the deep synovium and synovial fluid 15. These characteristics suggest that transdermal delivery may be an effective method for administering drugs to joints. However, the presence of the stratum corneum forms the major barrier to transdermal delivery. Microneedles (MNs) have recently emerged as a promising drug delivery strategy due to their excellent ability to penetrate the stratum corneum and their painlessness 16-18. Moreover, this platform has shown great promise for delivering proteins and antibodies while maintaining their bioactivity 19. From a clinical translation perspective, MN patches present a promising solution for large-scale manufacturing and offer the advantage of self-administration. This innovative approach not only alleviates the burden on patients but also significantly reduces healthcare costs, highlighting their remarkable potential for effective clinical application 20. However, there is a lack of understanding regarding the therapeutic mechanisms and outcome evaluation of MN-delivered De for OA, warranting further exploration.

In this study, we fabricated dissolvable MN patches loaded with De (MNs@De) and found that this approach has advantages in terms of attenuating knee OA in both rodents and Beagle dogs. Compared with local and systemic delivery, MN administration was better able to deliver De into the joint. Interestingly, single RNA sequencing revealed that senescence-associated secretory phenotype (SASP), a hallmark of cell senescence, was substantially upregulated in macrophages in the knee joints of an OA mouse model, and this was significantly attenuated by De. Further, immunofluorescence (IF) showed that De reduced expression of the senescence markers CDKN2A/P16 and CDKN1A/P21 21 in macrophages of an OA mouse model, suggesting a key role of macrophage senescence as evidenced by the effects of De. Mechanistically, conditioned medium from senescent macrophages induced the catabolism of chondrocytes associated with upregulation of proinflammatory and chondrocyte catabolic factors, which was abolished by De. Collectively, this study offered a new delivery method for De, using MNs to delay OA progression by attenuating macrophage senescence.

## Results

### Fabrication and characterization of MNs@De

**Figure 1:**
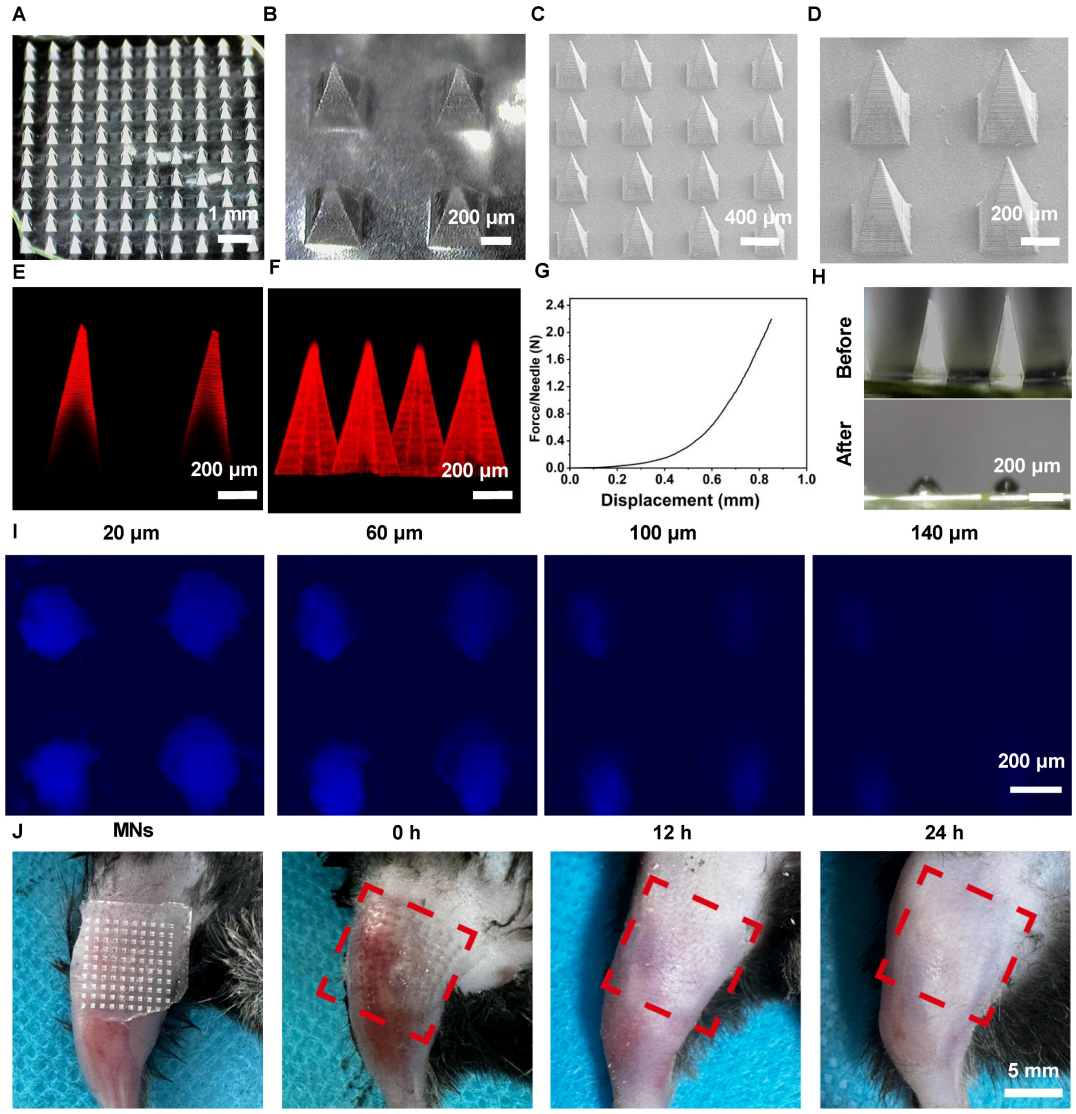
** Characterization of morphology and mechanical properties of denosumab (De)** (A, B) Dermoscopic images and (C, D) SEM images of the MNs@De patch. (E) Representative fluorescence image and (F) 3D image of the Cy5-labeled MNs@De patch. (G) Mechanical strength testing of the MNs@De patch. (H) Dermoscopic images of the MNs@De patch after application to porcine skin for 3 min. (I) Confocal laser scanning microscopy images of porcine skin at various depths after inserting the Cy5.5-labeled MNs@De patch. (J) Skin recovery after application of the MNs@De patch. Scale bar, 5 mm.

To study the therapeutic potential of MNs for the delivery of De in OA, we initially fabricated polyvinylpyrrolidone (PVP)-based MNs loaded with De using a two-step micro-molding process 22. PVP was identified as the optimal filling material for the MN tips, based on its superior mechanical strength and rapid dissolution rate. Moreover, due to its excellent compatibility, PVP has received approval from the Food and Drug Administration for use as a food additive and in pharmaceutical formulations 23. Dermatoscopy and scanning electron microscopy (SEM) were chosen to capture the morphology and architecture of MNs@De (Figure 1A-D). The results indicated that an MN patch consisting of a 10 × 10 array of needles exhibited a regular square-based pyramidal shape, with a height of 1,000 μm and a base diameter of 350 μm. Confocal laser scanning microscopy (CLSM) images showed that Cy5-labeled De was distributed in the needle tips, which enhanced its diffusion into the dermis and vascular network (Figure 1E, F). Next, the mechanical strength curve indicated that MNs@De have enough mechanical robustness to penetrate the stratum 24 (Figure 1G). After applying the MNs@De to pig skin for 3 min, complete dissolution of the needles was observed, signifying the MNs' capability for transdermal De delivery (Figure 1H). Furthermore, CLSM images demonstrated that the penetration depth of MNs@De in pig skin was up to 140 μm (Figure 1I). Meanwhile, application of MNs@De to the joints of mice resulted in a well-defined array pattern on the skin, with any MN-induced wounds healing within 12 h, leaving no damage to the skin (Figure 1J). Subsequently, we also used FITC-labeled BSA as a model drug to evaluate the drug release efficiency from the PVP MN. The results showed that the drug was completely released from the MNs within 3 min (Figure S1).

### The *in vivo* release behavior of MNs@De

**Figure 2:**
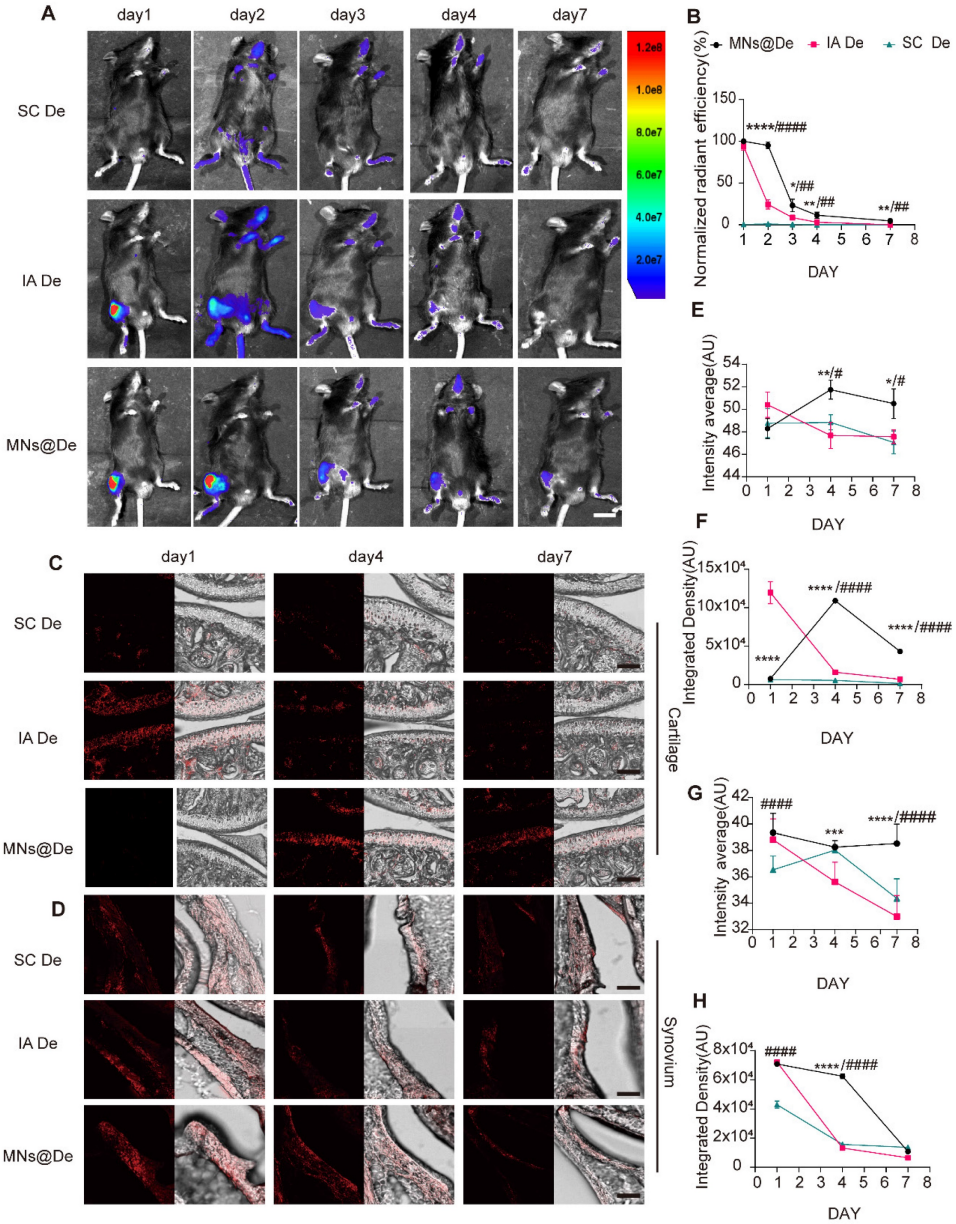
**The *in vivo* release behavior of dissolvable MN patches loaded with De (MNs@De).** (A) Representative images of Cy5.5-labeled De delivered via systemic subcutaneous injection (SC De), local injection (IA De), or microneedle injection (MNs@De) over 1-7 days. Scale bar, 1 cm. (B) Quantitative analysis of normalized time-course fluorescent radiant efficiency within the mouse knee joints over 7 days (n = 6 per group). The fluorescence radiant efficiency of the MNs@De group on day 1 was used as a control for the normalization. (C, D) Fluorescence images of mouse cartilage and synovium treated with Cy5.5-labeled De delivered by subcutaneous injection (SC), local injection (IA), or MN injection on days 1, 4, and 7. De (red, Cy5.5). Scale bars, 100 μm. (E-H) Quantitative analysis of the mean fluorescence intensity and total fluorescence intensity of De distribution in cartilage and synovium (n = 6 per group). Comparisons between the MN group and other groups were made using Student's t-test. *(MN vs. IA); #(MN vs. SC). Error bars represent mean ± SD. */#P < 0.05, **/##*P* < 0.01, ***/###* P* < 0.001, ****/####* P* < 0.0001.

MNs@De can deliver the drug in the periarticular area. To study its *in vivo* distribution, an imaging system assessed Cy5.5-labeled De retention in mouse joints after administration via three delivery methods: systemic injection (SC De), intra-articular injection (IA De), and MNs@De, over 7 days (Figure 2A). The formulation concentration was adjusted to ensure equal amounts of Cy5.5 in each group, with all groups receiving 20 μg De. For SC De, the weak fluorescent signal at the joint indicated its limited ability to deliver drugs to the joint. The IA De showed the strongest signal on day 1, but this decreased over time, weakening on day 3 and further reducing on day 4, indicating that the drug was metabolized over time and not effectively utilized. In contrast, drugs delivered by MNs penetrated the skin to reach the joint cavity. Unlike direct intra-articular injection, MN delivery had a sustained-release effect. *In vivo* imaging showed that MN-delivered drugs remained around the joint cavity for a longer period, enabled continuous drug delivery to the joint and achieved more durable therapeutic effects (Figure 2A, B).

To clarify the diffusion pattern of De delivered by MNs within the joint, mouse knee joints were harvested at 1, 4, and 7 days post-delivery, starting from 1 h post-delivery, along with a negative control group, and cryosections were prepared (Figure S2A). De was found to be mainly enriched in the synovium and cartilage (Figure 2C, D). In the cartilage, the overall fluorescence in the SC De group was not significant, while the IA De group had stronger fluorescence than the MNs@De group on day 1 (*P* < 0.0001). By day 4, the cartilage fluorescence in the IA De group had significantly decreased, while that in the MNs@De group had increased, showing stronger overall and mean fluorescence than the IA De group (*P* < 0.0001 and *P* < 0.01, respectively). By day 7, the MNs@De group still showed stronger fluorescence than the other two groups (*P* < 0.0001) (Figure 2C and E). In the synovium one day post-delivery, there was no difference in overall fluorescence between the IA De and the MNs@De groups, but both were stronger than the SC De group (*P* < 0.0001). By day 4, the MNs@De group outperformed the other two groups, and by day 7, there was no significant difference in fluorescence among the three groups (Figure 2D and F).

We attribute the sustained delivery effect from the MNs@De group to the diffusion time of De from the skin to the joint. To investigate how De passes through the soft tissue and enters the joint, we took cryosections of the skin and subcutaneous tissues of mouse knees after treatment with MNs@Vehicle (Veh) or MNs@De. The results showed that a large amount of drug residue remained in the skin 1 h post-delivery, but the fluorescence intensity significantly decreased and had disappeared by day 4 (Figure S2B).

Overall, the data suggest that MNs provide effective drug permeability for intra-articular drug delivery and promote sustained drug retention within the joint cavity due to the time required for drug diffusion.

### MNs@De alleviate the progression of OA in mice with good biosafety

**Figure 3:**
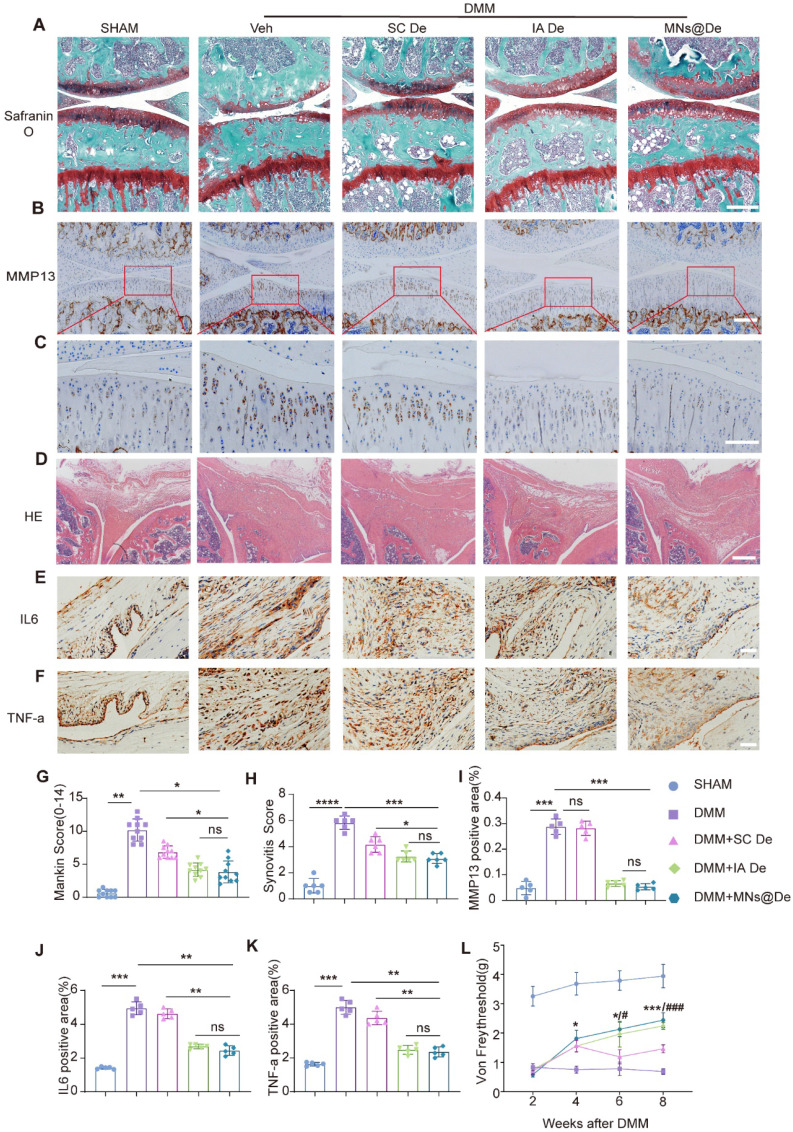
** MNs have similar therapeutic effects to local injection in the mouse OA model.** (A) Safranin O/Fast green staining of knee joints in sham, destabilization of the medial meniscus (DMM), SC De, IA De and MNs@De mice. Scale bar = 200 μm. (B, C) Corresponding quantification of MMP13 immunohistochemical (IHC) staining of the rat synovium and cartilage explants. (D) Hematoxylin and eosin (H&E) staining of synovium in sham, anterior cruciate ligament transection (ACLT), SC De, IA De and MNs@De rats. (E, F) Immunostaining of IL-6 and TNF-α in synovial membrane of sham, DMM, SC De, IA De and MNs@De mice. (G) The OA severity of knee joints was measured by Mankin score at 8 weeks after treatments (n = 10 per group). (H) Synovitis scores were measured at 8 weeks after treatments (n = 6 per group). (I) Corresponding quantification of MMP13 IHC staining of the mouse cartilage explants (n = 6 per group). (J, K) Corresponding quantification of IL-6 and TNF-α IHC staining of the mouse synovium explants (n = 5 per group). (L) Pain analysis was performed using von Frey assay according to paw withdrawal threshold (PWT) at 2, 4, 6 and 8 weeks after DMM surgery (n = 10 per group). Statistical analyses were conducted using one-way ANOVA followed by the Dunnett multiple comparisons test. Error bars are mean ± SD, * *P* < 0.05, **P < 0.01. Comparisons were made between groups with or without MNs@De treatments in mice following DMM.

To test the effects of MNs@De in OA, we performed destabilization of the medial meniscus (DMM) on the right hind limb knee joint of 12-week-old male mice, which is a well-characterized surgically induced rodent post-traumatic OA model. We first confirmed that both Veh and MNs@Veh have no therapeutic effects on OA (Figure S3). Then we divided the mice into five groups: sham, and then DMM treated with a PBS injection, with systemic SC De, with IA De, and with MNs@De. After 8 weeks of treatment, the knee joints were harvested for paraffin sectioning. OA progression was assessed through Safranin O & Fast Green staining and immunohistochemical (IHC) analysis. Consistent with previous findings, DMM-induced OA pathology was evident, with the MNs@De group demonstrating a Mankin score of 5.03 ± 1.87, indicating significant suppression of cartilage degeneration compared to systemic De treatment. Notably, the IA De group (Mankin Score: 5.07 ± 0.86) showed superior effects in delaying OA progression compared to SC De (IA De vs. SC De: *P* = 0.0405), while MNs@De exhibited comparable efficacy with IA De (MNs@De vs. IA De: *P* = 0.8149). The SC De group displayed mild cartilage erosion (7.18 ± 1.34), whereas untreated controls developed severe cartilage damage extending to the calcified zone (10.24 ± 2.86) (Figure 3A, G). Matrix metalloproteinase-13 (MMP13) expression was markedly elevated in untreated controls, whereas the sham surgery, MNs@De, and IA De groups showed comparable MMP13 levels (Figure 3B, I).

Given the pivotal role of synovitis in OA progression, synovial inflammation was evaluated using hematoxylin and eosin (H&E) staining and IHC according to OARSI histopathological guidelines 25. All experimental groups except sham controls exhibited synovial hyperplasia, with untreated animals showing substantial inflammatory cell infiltration in the synovial layer (7-fold increase vs. sham, *P* < 0.0001). Both MNs@De and IA De groups demonstrated comparable synovitis scores (*P* > 0.05) and significantly reduced interleukin (IL)-6 and tumor necrosis factor alpha (TNF-α) expression in synovial tissues (Figure 3E, F), indicating potent anti-inflammatory effects of localized delivery approaches.

Pain is the primary symptom and main complaint of OA patients in clinical practice. We assessed pain levels using the von Frey test. We found that the paws of DMM mice were highly sensitive to stimuli, unlike those in the sham surgery group. All three treatment groups initially showed pain responses 2 weeks post-DMM surgery, likely due to postoperative pain. However, 4 weeks post-surgery, MNs@De treatment significantly increased the paw withdrawal threshold (PWT), and at 8 weeks post-surgery, the PWT in the MNs@De group was significantly higher than in the SC De group (*P* < 0.001), indicating that MNs@De effectively alleviates OA-related pain (Figure 3L).

To assess the potential systemic side effects of MNs in mice, we performed histopathological analysis on the main visceral organs of these mice. Compared with mice that did not receive any treatment, we found no obvious tissue damage in any major organs of mice treated with MNs (Figure S4A). Routine hematological indicators of liver and kidney function were within the normal range (Figure S4B). Similarly, mouse marrow harvested after 8 weeks of MNs@De application revealed no significant alterations in immune-cell profiles (IHC for CD3, CD4, CD8 and CD20 Figure S5). These results reveal that the use of MN-mediated OA drug treatment has good biosafety and almost no systemic toxicity.

### MNs@De relieve the progression of synovitis in a rat OA model induced by intra-articular injection of monoiodoacetate (MIA)

**Figure 4:**
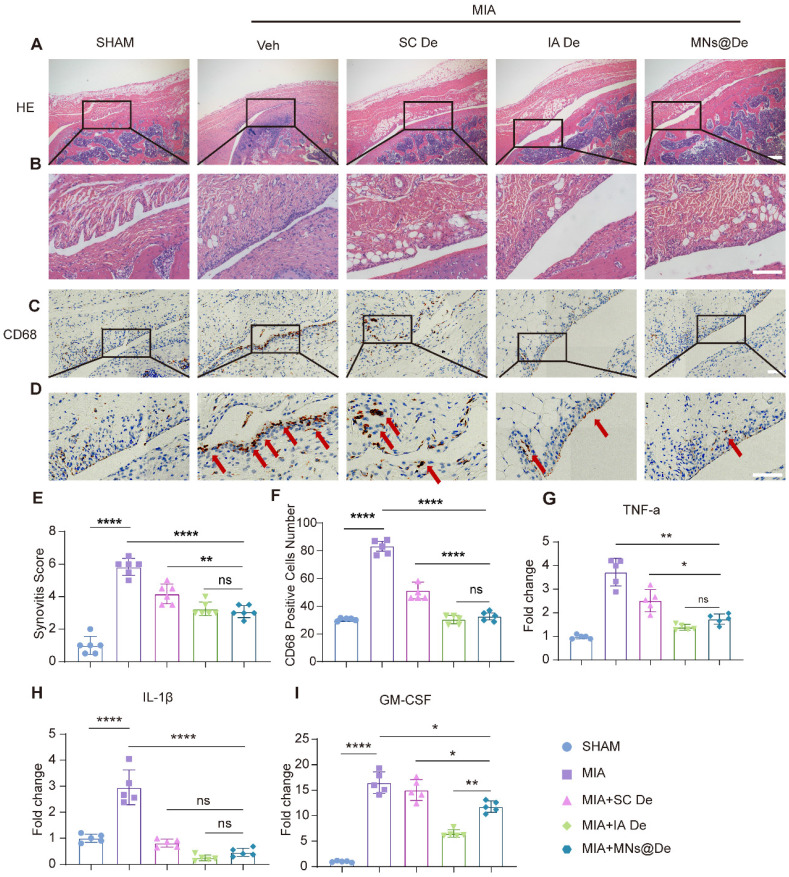
** In the treatment of inflammatory (MIA) arthritis model SD rats, all three treatment methods had an effect on synovitis, and the microneedle group was not inferior to the local injection group.** (A, B) H&E staining of synovium in sham, MIA, SC De, IA De and MNs@De rats. (C, D) Immunostaining of CD68 (red arrows) in synovial membrane of sham, MIA, SC De, IA De and MNs@De rats. Scale bar, 100 μm. (E) Synovitis scores were measured at 4 weeks after treatments (n = 6 per group). (F) The numbers of CD68^+^ cells within synovial membrane (n = 5 per group). (G, H, I) Relative gene expression of TNF-α, IL-1β and GM-CSF in rat synovium explants was examined by quantitative real-time polymerase chain reaction (qRT-PCR) (n = 5 per group). Scale bar, 100 μm. Statistical analyses were conducted using one-way ANOVA followed by the Dunnett multiple comparisons test. Error bars are mean ± SD, * P < 0.05, **P < 0.01, ***P < 0.001, ****P < 0.0001. Comparisons were made between groups with or without MNs@De treatments in rats with MIA.

As the RANK/RANKL pathway plays a key role in inflammation, and De inhibits synovitis, we therefore explored the effects of De on inflammatory arthritis by intra-articular injection of MIA 26. One day after MIA injection, rats were randomly divided into four groups: PBS injection group, SC De group, IA De group, and MNs@De group. After one month of treatment, we collected knee joints from each group for paraffin sectioning. To assess the progression of synovitis, we used H&E staining and IHC to evaluate synovial inflammation. The untreated group showed a significant increase in the number of synovial lining cells, synovial tissue hyperplasia, and a large infiltration of inflammatory cells (Figure 4A, B), probably macrophages 27, which was significantly blocked by De, especially in the IA De and MNs@De groups. We then performed IHC using the common macrophage marker CD68 and found that CD68^+^ macrophages were highly expressed in the untreated group, which was obviously inhibited by systemic De injection. However, the effects in the MNs@De group and the IA De group were stronger than in the SC De group (Figure 4C, D).

Additionally, we collected synovial tissue samples from MIA-treated rats for RT-qPCR analysis. Results showed that in the MNs@De group, IL-1β RNA levels were significantly reduced by approximately 65% (*P* < 0.01) and TNF-α RNA levels by over 65% (*P* < 0.0001) compared to the untreated group. No significant differences were found between the MNs@De and IA De groups. Moreover, IL-1β and granulocyte-macrophage colony-stimulating factor (GM-CSF) RNA levels in the MNs@De group remained lower than in the SC De group (*P* < 0.05). However, GM-CSF RNA levels were significantly lower in the IA De group than in the MNs@De group (*P* < 0.01) (Figure 4I). Therefore, we concluded that De alleviates the symptoms of synovitis induced by MIA in rats, with the MNs@De group showing the best therapeutic effect.

### De ameliorates chondrocyte catabolism by alleviating macrophage senescence

We previously found that De is distributed in cartilage after treatment (Figure 2C). We then looked at the direct effects of De on chondrocytes. Surprisingly, when we performed PCR analysis we found no changes in the expression levels of metabolic factors after treating chondrocytes with De, suggesting that De may have no direct effects on chondrocytes (Figure S6). As De mainly targets monocyte-macrophage lineage cells, and we found a substantial infiltration into the synovium, which was abolished by De (Figure 4C), we next explored how De modulates the function of macrophages and delays OA. RAW 264.7 cells were stimulated with IL-1β (10 ng/mL) and divided into three groups: PBS control, De monotherapy (De), and IL-1β + De co-treatment (IL-1β+De). Transwell migration assays revealed that IL-1β stimulation significantly increased macrophage migration to the lower chamber, while De treatment significantly reduced those effects (Figure S7A, B). CCK-8 assays demonstrated that IL-1β-treated cells exhibited markedly elevated proliferation at 24 h, peaking at 72 h. De monotherapy showed the lowest cell count at 24 h and the slowest proliferation rate, whereas IL-1β+De restored proliferation to baseline (Figure S7C). These findings suggested that De suppressed macrophage proliferation and migration. Moreover, western blot analysis showed significantly upregulated arginase-1 (ARG1) and obviously downregulated CD86 and TNF-α expression in De-treated macrophages (Figure S7D), indicating anti-inflammatory effects of De on macrophages.

Given the close association between inflammation and senescence 28, we first explored the role of inflammation in macrophage senescence. SA-β-gal staining revealed elevated senescent (SA-β-gal⁺) macrophages in inflammatory conditions, which were reduced by De. De monotherapy also slightly decreased SA-β-gal⁺ cells compared to controls (Figure S7E). Western blotting confirmed increased p16 and p21 levels in untreated inflammatory macrophages, which were normalized by De (Figure S7D). These data suggest that De alleviates senescence induced by inflammation.

**Figure 5:**
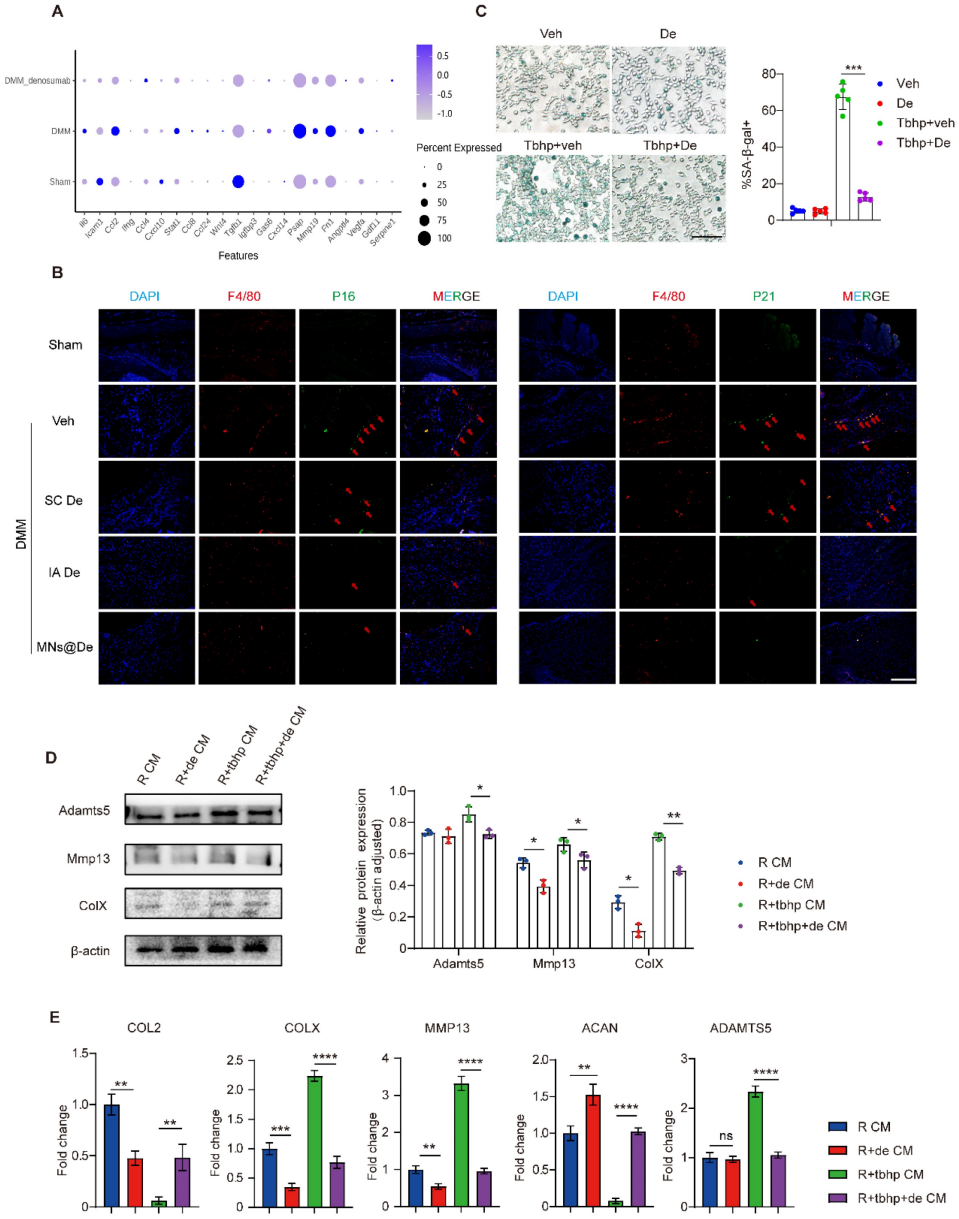
** Denosumab ameliorated chondrocyte phenotype by alleviating macrophage senescence.** (A) mRNA sequencing was performed to analyze the SASP gene set, with results presented as a bubble plot. The groups included sham, DMM, and DMM + De (n = 6 per group). (B) P16, P21 (red arrows), and F4/80 were stained using immunofluorescence; nuclei were counterstained using DAPI; representative images are shown; scale bar, 200 μm. (C) SA-β-gal staining of macrophages induced to senescence by TBHP and semi-quantitative analysis, scale bar, 200 μm (n = 5 per group). (D) Western blot analysis of the protein levels of ADAMTS5, MMP13, and COLX in chondrocytes treated with macrophage-conditioned medium, quantification of protein expression was performed using ImageJ software (n = 3 per group). (E) RT-qPCR was performed to evaluate COL2, COLX, MMP13, ACAN, and ADAMTS5 mRNA levels in chondrocytes treated with macrophage-conditioned medium (n = 5 per group). Statistical analyses were conducted using one-way ANOVA followed by the by Dunnett multiple comparisons test. Error bars are mean ± SD, * P < 0.05, **P < 0.01, ***P < 0.001, ****P < 0.0001.

Next, we wondered whether senescence plays a role in the effects of De on macrophages in OA. We first analyzed the phenotypical changes of macrophages by single-cell sequencing, and found that the SASP gene set is upregulated in OA mouse knees (including IL-6, CCL2, MMP19, PSAP, and Fn1), but this is downregulated by De (Figure 5A), and this was further confirmed by PCR analysis (Figure S8A). Moreover, IF showed that there were more senescent macrophages in OA knee synovium, and this increase was greatly alleviated by De treatment. We performed IF staining for p16, p21, and F4/80 on synovial tissues from mice. The results showed significant expression of p16 and p21 in the DMM group, which colocalized with F4/80. In the SC De group, p16 and p21 expression were both slightly reduced, while the IA De and MNs@De groups exhibited significantly lower expression compared to the SC De group (Figure 5B and S9). These data clearly showed a strong association of macrophage senescence and OA treatment with De.

To explore the effects of De on macrophage senescence and thus OA, we first induced macrophage senescence by introducing tert-butyl hydroperoxide (TBHP) 29 and performed SA-β-gal staining. The results showed that the number of SA-β-gal⁺ macrophages significantly increased in the TBHP-treated group, indicating successful induction of cellular senescence, which was reduced by De treatment (Figure 5C).

Next, we explored how macrophage senescence modulates OA and the effects of De on this modulation. We collected the supernatants of the macrophages for ELISA to assess the expression of cartilage-damage factors (MMP13, MMP9, ADAMTS5) and inflammatory factors (IL-1β, IL6). Strikingly, all these factors were substantially upregulated by senescent macrophages, while they were greatly inhibited by De, to levels very similar to those of normal macrophages (Figure S8B). Conditioned medium from these macrophages was then used to treat chondrocytes and assess changes in the chondrocyte phenotype. Four groups were tested: normal macrophage-conditioned medium (R CM), De-treated macrophage-conditioned medium (R + De CM), TBHP-induced senescent macrophage medium (R + TBHP CM), and TBHP + De-treated macrophage medium (R + TBHP + De CM). Western blot analysis showed that the expression of catabolic proteins (ADAMTS5, MMP13, and COLX) was upregulated in the R + TBHP group. In the R + De CM group, ADAMTS5 expression was similar to the normal group, while MMP13 and COLX expression were both lower. The R + TBHP + De CM group exhibited normalized expression levels of these proteins (Figure 5D). Additionally, qPCR analysis of chondrocyte RNA showed that in the R + TBHP CM group, the expression of anabolic genes (COL2 and ACAN) was significantly reduced, while catabolic genes (COLX, MMP13, and ADAMTS5) were upregulated. In the R + TBHP + De CM group, expression of these genes was normalized, and the R + De CM group showed higher ACAN expression and lower COLX and MMP13 expression compared to the normal group (Figure 5E). These findings indicate that macrophage senescence induces chondrocyte catabolism, which is abolished by De, thereby ameliorating OA.

### MNs@De reduce cartilage wear in a large animal (Beagle) OA model

**Figure 6:**
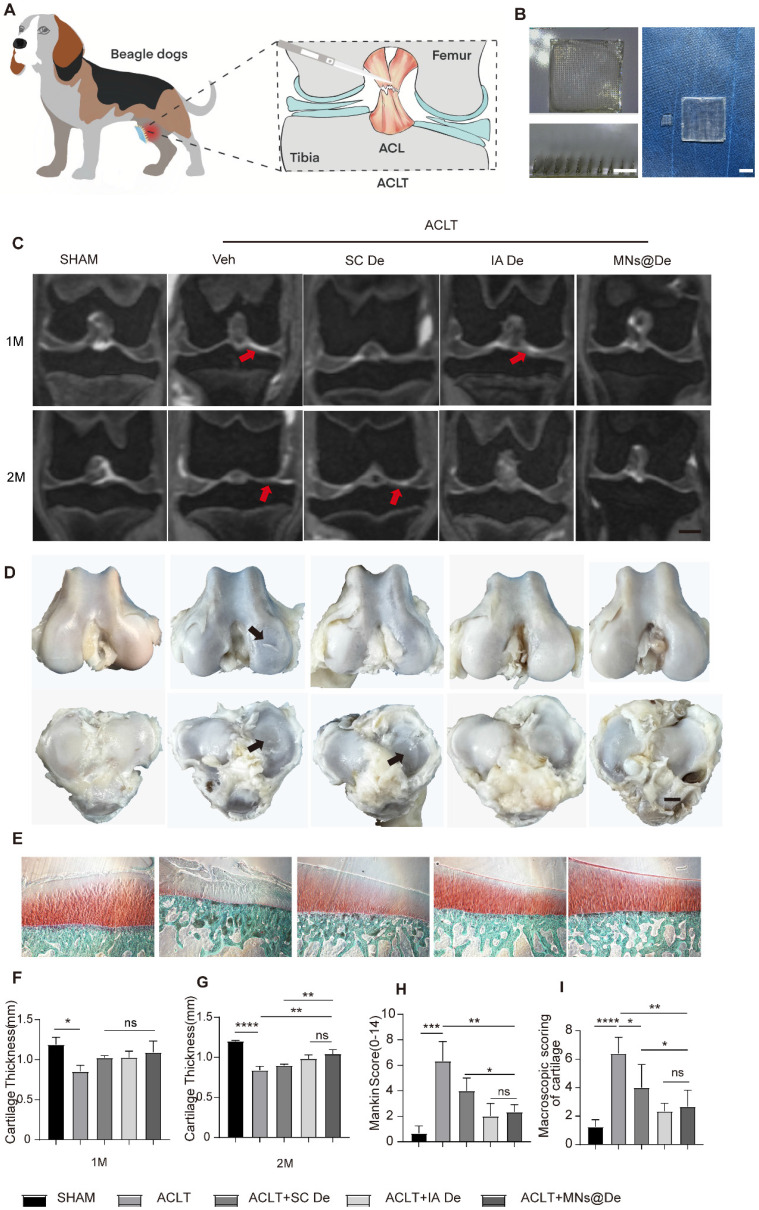
**The effect of microneedles in alleviating cartilage wear in a large animal (Beagle) OA model.** (A) Schematic diagram of ACLT surgery in Beagle dogs. (B) Visual representation of the large microneedles (3 cm × 3 cm) used in this experiment, alongside a comparison with the smaller microneedles (1 cm × 1 cm). Scale bar, 1cm. (C) Macroscopic appearance of the femoral condyles and tibial plateau cartilage after two months of indicated treatment. Black arrows indicate cartilage damage. Scale bar, 5 mm. (D) MRI coronal images of the Beagle dogs at one month and two months post-treatment to assess cartilage thickness and damage. Red arrows indicate cartilage damage. Scale bar, 1 cm. (E) Safranin O/Fast green staining of tibial plateaus in sham, ACLT, SC De, IA De and MNs@De Beagles. Scale bar, 200 μm. (F) The OA severity of tibial plateaus was measured by Mankin score at 8 weeks after treatments (n = 5 per group). (G) Macroscopic cartilage scoring of the four main weight-bearing areas: the medial femoral condyle, medial tibial plateau, lateral femoral condyle, and lateral tibial plateau, based on the OARSI scoring system (n = 5 per group). (H, I) Quantification of the medial tibial plateau joint cartilage thickness in the coronal plane using MRI images (n = 5 per group). Statistical analyses were conducted using one-way ANOVA followed by Dunnett's multiple comparisons test. Error bars are mean ± SD, * P < 0.05, **P < 0.01, ***P < 0.001, ****P < 0.0001. Comparisons were made between groups with or without MNs@De treatments in beagles with ACLT.

Based on the therapeutic efficacy of De observed *in vitro* and *in vivo* in mice, we used anterior cruciate ligament transection (ACLT)-induced OA in the Beagle dog knee as a large animal model, which is more comparable to humans. We selected male Beagle dogs (10 kg) for the ACLT surgery (Figure 6A), and treatments were administered once per month postoperatively, with groups divided into PBS injection, SC De, IA De, and MNs@De groups. Due to the larger size of the animals, the dose of De was increased from the original 20 μg to 2 mg based on body weight. To facilitate clinical translation of De-loaded MNs, we engineered large-scale patches (3 cm × 3 cm) with optimized structural integrity, and electron microscopy demonstrated a 1.3-3.3% tip defect rate (Figure 6B and Figure S10A-F). The patches exhibited superior penetration efficiency (92-97% through five-layer Parafilm M barriers) and rapid needle dissolution (within 3 min), confirming that the bigger MN patches showed sufficient mechanical strength for transdermal drug delivery (Figure S10G, H).

To assess the effect of De on cartilage erosion, we used *in vivo* MRI to dynamically assess pathological changes in cartilage. Coronal MR images of the Beagle knee joints revealed that at one month postoperatively, the cartilage thickness in the untreated group (0.85 ± 0.17 mm) was reduced compared to the sham surgery group (1.19 ± 0.18 mm) (*P* < 0.05), but there were no significant differences among the three treatment groups, nor within the groups themselves. At two months postoperatively, the cartilage thickness in the untreated group (0.83 ± 0.11 mm) was significantly reduced compared to the sham surgery group (1.20 ± 0.0216 mm) (*P* < 0.0001), and both IA De (0.98 ± 0.08 mm) and MNs@De (1.04 ± 0.09 mm) effectively mitigated the degree of cartilage wear, superior to systemic subcutaneous treatment (0.89 ± 0.03 mm) (*P* < 0.01) (Figure 6C, F, and G).

After two months of treatment, the knee joints of the Beagles were harvested, and their gross appearance was evaluated according to the canine OA scoring system published by the OARSI Histopathology Initiative 30. The untreated group and the SC De group showed more pronounced cartilage wear. In contrast, the MNs@De and IA De groups exhibited smoother surfaces (Figure 6D and I). We used Safranin O and Fast Green staining for histological analysis to observe the pathological changes in the tibial plateau cartilage of Beagle dogs. The untreated group (Mankin score: 6.33 ± 1.53) showed significant cartilage damage and proteoglycan loss. The SC De group (Mankin score: 4.10 ± 1.18) showed a mitigating effect on cartilage wear, but this treatment was less effective than in the IA De group (Mankin score: 2.06 ± 0.91) or the MNs@De group (Mankin score: 2.33 ± 0.58). The latter two groups maintained almost the same cartilage structure as the sham-operated group, effectively alleviating cartilage wear (Figure 6E and H). These data indicate that the MNs@De group still has the advantage of delaying cartilage wear in large animal models compared to systemic injection.

### MNs@De alleviate the progression of synovitis in a large animal (Beagle) OA model

**Figure 7:**
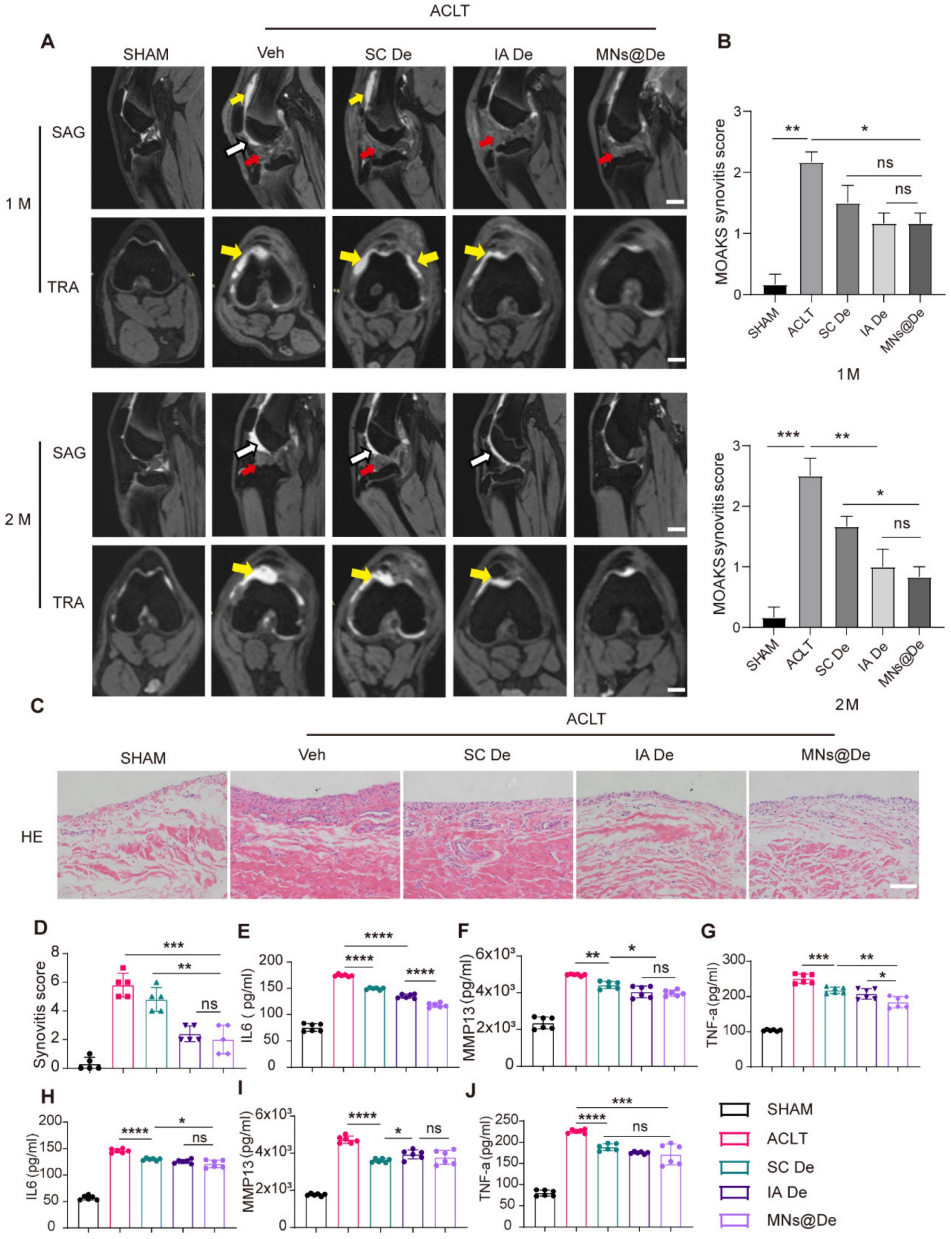
**The effect of microneedles in alleviating inflammation in a large animal (Beagle) OA model.** (A) Sagittal and transverse MRI images of Beagle dogs following sham surgery or ACLT at 1 month and 2 months postoperatively. Scale bar, 1 cm. (B) The MOAKS of synovitis in Beagle dogs following sham surgery or ACLT at 1 month and 2 months postoperatively (n = 5 per group) (C) H&E staining of synovium in sham, MIA, SC De, IA De and MNs@De Beagles. Scale bar, 200 μm. (D) Synovitis scores were measured at 8 weeks after treatments (n = 5 per group). (E-J) The concentration of pro-inflammatory cytokines (IL-6 and TNF-α) and collagenase (MMP13) in knee synovial fluid and blood serum after 2 months of treatments, as assessed by enzyme-linked immunosorbent assay (n = 6 per group). Statistical analyses were conducted using one-way ANOVA followed by the Dunnett multiple comparisons test. Error bars are mean ± SD, * P < 0.05, **P < 0.01, ***P < 0.001, ****P < 0.0001. Comparisons were made between groups with or without MNs@De treatments in Beagles following ACLT.

The MOAKS (MRI Osteoarthritis Knee Score) scoring system is a semi-quantitative MRI scoring tool used to assess knee OA 31. It was developed based on the Whole Organ Magnetic Resonance Imaging Score (WORMS) and the Boston-Leeds Osteoarthritis Knee Score (BLOKS) scoring tools and has been proven to have very good to excellent reliability 32. We used the MOAKS score to analyze the synovitis condition in Beagle dogs one and two months after ACLT surgery based on their MRI images (sagittal and coronal sections). In the figures, yellow arrows indicate joint effusion, and white arrows indicate hardening of the fat pad (Figure 7A). One month postoperatively, we found that all groups except the sham surgery group showed hardening of the fat pad, but the untreated group still had a large amount of effusion into the joint cavity, while the SC De and IA De groups showed only a small amount of effusion, and there was no statistically significant difference in MOAKS scores among the three treatment groups (Figure 7B). Two months postoperatively, the untreated group (MOAKS score: 2.50 ± 0.50) had more effusion and continued hardening of the fat pad, while in the SC De group (MOAKS score: 1.67 ± 0.29), joint effusion was reduced but the hardening of the fat pad persisted. In both the IA De group (MOAKS score: 1.07 ± 0.51) and the MNs@De group (MOAKS score: 0.83 ± 0.29), no hardening of the fat pad was visible, but in the MNs@De group, there was less joint effusion and a clearer joint tissue structure. We used H&E staining for histological analysis to observe the pathological changes in the synovium of Beagle dogs. In both the untreated group and the SC De group, the synovial interstitium was partially thickened with angiogenesis, and the synovial cell layer in the untreated group was significantly thickened with a large infiltration of inflammatory cells, while in the IA De group and the MNs@De group the synovium did not thicken and retained the same morphology as the sham surgery group (Figure 7C). Additionally, we further analyzed release of the pro-inflammatory cytokines IL-1β and TNF-α, and the cartilage erosion factor MMP13 in joint fluid (Figure 7E-G) and serum (Figure 7H-J) to assess the severity of the inflammatory response. Consistent with our observations of the MRI images, cytokine release in the MNs@De group was lower than in the other groups.

These data support the application of MNs@De as a long-term drug delivery method that delays the progression of synovitis and OA in a preclinical canine OA model.

To assess the potential systemic side effects of MNs on large animals, such as beagle dogs, we conducted a histopathological analysis of their major visceral organs. Compared to the Beagles that received no treatment, we found no significant tissue damage in any major organs of the Beagles treated with MNs (Figure S11). These results reveal that the use of MN-mediated OA drug treatment possesses good biosafety and causes minimal systemic toxicity in large animals.

## Discussion

We developed dissolvable MN patches loaded with De (MNs@De) and achieved long-term retention of De around joint cavities. Tests in various OA animal models confirmed their therapeutic effects. MNs@De significantly reduced cartilage degeneration, synovitis, and pain, proving to be more effective than traditional systemic injections while being comparable to intra-articular injections. Mechanically, OA triggers macrophage senescence, leading to increased expression of pro-inflammatory and cartilage-damaging factors, changes which are inhibited by De.

A major challenge in OA therapy is the lack of disease-modifying treatments, as current treatments only address symptoms like pain and inflammation without altering disease progression. De, effective in osteoporosis, works by inhibiting activity of monocyte-macrophage-osteoclast lineage cells, reducing bone resorption and inflammation.

In OA, abnormal activation of these cells increases local inflammation, cartilage damage, and subchondral bone remodeling-all critical OA features. Notably, a recent study has emphasized that systemic administration of De delays the progression of hand OA 7, indicating its structure-modifying effects by inducing abnormal bone remodeling and preventing new erosive joint formation in erosive hand OA. However, knee OA differs significantly from hand OA in anatomy, biomechanics, and pathological heterogeneity, making knee OA treatment more challenging. Interestingly, single-cell sequencing analysis revealed upregulated expression of mechanosensitive genes such as Piezo1 and TRPM7 in OA macrophages, which was downregulated by De (data not shown). This suggests that De may modulate the interplay between biomechanical signaling and cellular senescence in OA pathogenesis.

Traditional administration methods such as systemic and intra-articular injections also pose several challenges. Systemic injections may lead to suboptimal drug distribution and systemic side effects, while intra-articular injections, although they increase local drug concentration, are invasive and carry infection risks. To tackle these challenges, we explored the benefits of MN drug delivery. The PVP and PVA materials we used are FDA-approved and show great potential for clinical use. MNs can evenly deliver De to the periarticular area, ensuring its uniform distribution in cartilage and synovial tissue, enhancing drug retention in the joint. This uniform distribution and drug retention mean that MNs@De are more effective than traditional systemic injections and their effectiveness matches intra-articular injections. Imaging showed that MNs@De maintained stronger fluorescence than intra-articular injections over seven days (Figure 2A, B), proving their drug retention ability. Cryosection analysis revealed that De accumulated in synovial and cartilage tissue and increased over four days (Figure 2C, D), confirming sustained drug presence. In various animal models, MNs@De showed remarkable therapeutic effects. In DMM mice, the Mankin score of the MNs@De-treated group was 5.03 ± 1.87, lower than the group treated with systemic injections (7.18 ± 1.34, *P* = 0.0405) and similar to those receiving intra-articular injections (5.07 ± 0.86, *P* = 0.8149) (Figure 3A, G). In MIA-induced rats, MNs@De reduced IL-1β and TNF-α RNA levels by approximately 65% (*P* < 0.01 and *P* < 0.0001, respectively) (Figure 4G, H).

To further verify the efficacy of MNs@De in treating OA, we conducted experiments on a large animal model-the Beagle dog. Beagle dogs have joint structures and physiological environments similar to humans, making them a good model for simulating human OA conditions compared with rodents. The results showed that MNs@De significantly slowed cartilage erosion. Specifically, the cartilage thickness in the MNs@De group was 1.04 ± 0.09 mm at two months post-surgery, which was significantly greater than that in the untreated group (0.83 ± 0.11 mm, *P* < 0.0001) or the systemic injection group (0.89 ± 0.03 mm, *P* < 0.01) (Figure 6F, G). Moreover, using the MOAKS to evaluate synovitis, the MNs@De group had a MOAKS of 0.8333 ± 0.2887, which was significantly lower than the untreated group (2.50 ± 0.50, *P* < 0.01) or the systemic injection group (1.67 ± 0.29, P < 0.05), and comparable to the intra-articular injection group (1.07 ± 0.51) (Figure 7A, B). These findings indicate that MNs@De also have a significant advantage in reducing synovitis.

Further mechanistic studies have unveiled the potential mechanism by which De ameliorates OA. De inhibits macrophage senescence, reducing their secretion of inflammatory and cartilage-damaging factors, which indirectly harm chondrocytes (Figure 5D, E), a new mechanism of De in treating OA. Cellular senescence, a key cause and intervention target of inflammatory responses and tissue damage 33, is characterized by permanent cell-cycle arrest and secretion of tissue-damaging and inflammatory factors into the microenvironment, known as the SASP. This phenotype contributes to chronic tissue-damaging diseases such as pulmonary fibrosis, atherosclerosis, and arthritis 34, 35. Thus, inflammation and tissue damage are important pathological features of senescence 36, 37. Studies have shown that an increase in senescent cells in joints is closely related to OA progression 38. Clearing these cells slows OA progression, possibly due to the high expression of SASP factors (including IL-1, IL-6, and MMP19) in senescent cells, which promote inflammatory responses and tissue damage 39, 40. Macrophage senescence-induced systemic inflammation and tissue damage are important mechanisms leading to multi-tissue aging diseases 41, such as osteoporosis 42, 43. Inhibiting macrophage pyroptosis to clear senescent joint cells can alleviate OA 44. However, the direct role and mechanism of macrophage senescence in OA have not been reported. We first identified upregulated synovial macrophage senescence in OA mice and the inhibitory effect of De on this phenotype (Figure 5A, B, C) using single-cell sequencing and IF. Further mechanistic studies confirmed that senescent macrophages secrete various inflammatory and cartilage-damaging factors, some of which are also characteristic of the SASP, such as ILs and MMPs, increasing chondrocyte catabolism, and that this effect can be blocked by De (Figure 5D, E). This mechanism may involve RANKL-induced upregulation of downstream NF-κB, promoting cellular senescence 45, 46, which could be inhibited by De. These results indicate that De effectively improves the pathological features of OA by inhibiting macrophage senescence and the secretion of proinflammatory and catabolic factors. These findings validate the effectiveness and superiority of MNs@De across different animal models and provide key scientific support for their clinical translation.

While our study achieved positive results in animal models, translating these findings to human clinical applications poses challenges. First, as the study was predominantly conducted in animal models, it is crucial to successfully translate these findings to human clinical trials. Future research should evaluate the pharmacokinetics, safety, and long-term efficacy of MNs@De in humans.

Additionally, the MN materials currently used, such as PVP and PVA, have good biocompatibility 47, 48 but offer room for improvement in delivery depth and efficiency. For instance, MNs can only deliver De to a skin depth of approximately 200 μm, after which the drug must passively diffuse into the joint cavity, risking retention in non-target tissues. Future studies might explore combining MNs with gas-propulsion technologies—using oxygen 49, hydrogen 50, or carbon dioxide 51—to enhance delivery depth and efficiency, optimizing therapeutic outcomes. By addressing these limitations, MNs@De show promise as a safer and more effective OA treatment, potentially improving clinical outcomes for patients.

## Materials and Methods

### The fabrication process of dissolvable MN patches loaded with De

First, MN mater templates of different sizes were fabricated using 3D printing for application in rat and dog models. The small MN mater template consisted of a 10 × 10 array, each needle with a 350 µm base diameter and a 1000 µm height, forming a pyramid structure. The inter-needle tip spacing was 700 µm. The large MN mater template consisted of a 33 × 33 array, each needle with a 450 µm base diameter and a 1000 µm height, forming a pyramid structure. The inter-needle tip spacing was 900 µm. The representative PDMS mold was fabricated by replicating the 3D-printed MN master templates.

MNs@De patches (small, used on rodent models) were prepared using a two-step micro-mold method. PVP and De were dissolved in dd-water to prepare the mixture (50 mg/mL, PVP:De = 20:1). Subsequently, this mixture was poured into the PDMS mold, vacuumed for 15 min, and then sealed for overnight drying. A PVP/PVA solution (20%, PVP:PVA = 5:1) was cast onto the mold and dried overnight. Finally, the DS MN patches (small) were stripped and stored for future use.

MNs@De patches (large, used on Beagle dogs) were prepared using a similar procedure to that described above. PVP and De were dissolved in dd-water to prepare the mixture (100 mg/mL, PVP: De = 5: 1).

The morphological evaluation and penetration capability of the macro-scale MNs were systematically investigated. Dimensional measurements of the MN patch were initially performed using calibrated rulers, followed by electron microscopy (ML31, Guangzhou Mshot Photoelectric Technology Co. Ltd.) to characterize tip morphology, base configuration, and critical geometric parameters (tip height, base dimensions, and apex width). Mechanical robustness was assessed through penetration tests using a five-layer Parafilm M membrane model. Dissolution kinetics were evaluated by immersing the MN patch in a hydrogel matrix for 3 min, with subsequent microscopic examination of tip degradation patterns.

### Animals

Male C57BL/6J mice at 12 weeks old or Sprague-Dawley (SD) rats (weight 200-225g) were used as experimental animals which were purchased from Beijing SPF Biotechnology Co. Ltd. Twelve male canines (Beagle), with a mean body weight of 10.0 ± 1.50 kg, were purchased from Wuhan Xinzhou Wanqianjiahe Experimental Animal Breeding Farm Co. Ltd. and housed on the first floor of the Huazhong University of Science and Technology Tongji Medical College Laboratory Animal Centre in an open environment. The Animal Care and Ethics Committee at Huazhong University of Science and Technology approved all animal experiments related to this study (Ethical approval number: 4067).

### OA induction and treatment

DMM surgery was used to create an OA mouse model, as previously described 52. In brief, mice were anesthetized with pentobarbital sodium (50 mg/kg), and a longitudinal skin incision was made on the medial side of the right knee joint. Under a surgical microscope, the medial meniscal tibial ligament was transected. Control mice underwent the incision without transection of the meniscal tibial ligament.

For the traumatic SD rat model, OA modelling was performed by anterior cruciate ligament transection (ACLT) 53. After anesthesia with pentobarbital sodium, the knee joint skin was incised along the patellar ligament, and the patella was moved to one side to expose the knee joint cavity in a flexed position. The anterior cruciate ligament could then be clearly visualized, transected, and a drawer test was performed to verify. The control group received an incision without transection of the anterior cruciate ligament. An SD rat model of inflammatory OA was induced with mono-iodoacetate (MIA; Sigma, St Louis, MO, USA). In brief, rats were anesthetized with pentobarbital sodium and received a single intra-articular injection of 3.0 mg/50 μL MIA 54 (in PBS) into the right knee joint using a 31G insulin syringe. The control group received an equal volume of 0.9% sterile saline.

As for dogs, OA was induced by ACLT surgery 55. After anesthesia with an intramuscular injection of Su Tai (10 mg/kg), the skin was incised along the medial side of the patellar ligament with a scalpel, then bluntly dissected to the joint capsule at the surface of the joint cavity. After opening the joint capsule with curved scissors and a scalpel, the anterior cruciate ligament of the right knee joint was cut with curved scissors and a drawer test was performed for verification. After the resection, the synovium, fascia, and skin layers are all sutured. The contralateral knee joint served as an internal control. For the first three days after surgery, Beagle dogs received analgesics and antibiotics. Starting from the second day after surgery, Beagle dogs were driven in the cage for 10 min every day to promote activity.

### Use of MN patches to treat traumatic and inflammatory OA models

C57BL/6J mice and SD rats were divided into five groups (normal group, model group, systemic subcutaneous injection group, local injection group, and MN group). After anesthetizing the animals with pentobarbital, the MN patches were applied to the skin surface of the knee joint of the mice or rats, and after pressing with a thumb for 5-10 min, the MN patches were removed. It was observed that the tips of the MNs disappeared, indicating that the drug was completely dissolved into the subcutaneous tissue. In the local injection group the drug was directly delivered into the joint cavity through a syringe, while in the systemic subcutaneous injection group the thicker subcutaneous tissue at the back of the neck of the mice or rats was chosen for injection.

### OA pain analysis

Mouse knee joint pain was measured using von Frey fibers at 0, 2, 4, and 8 weeks after DMM surgery. Before the test, each mouse was acclimated on an individual 4 × 3 × 7 cm metal grid (Shanghai Yuyan Instrument Co. Ltd., Shanghai, China) for 20 min. Von Frey fibers ranging from 0.008 to 300 g were used for this assay. According to the "up-down method", the plantar surface of each mouse's hind paws was stimulated with each fiber until the fiber bent for 3 seconds. X indicates a withdrawal response, and O indicates no response. The formula shows that the threshold force causing paw contraction is expressed as the 50% paw withdrawal threshold: (10[Xr + kδ])/10,000 (Xr = value of the last von Frey fiber used in the sequence, expressed in logarithmic units; k = table value; δ = mean difference in forces between fibers).

### Histology and IHC

After 8 weeks of model establishment, animal knee joints were collected and immersed in a 4% formaldehyde solution overnight. Subsequently, decalcification was performed in 10% ethylenediaminetetraacetic acid (EDTA) for 2-3 weeks for mice and for one month for SD rats, followed by embedding in paraffin. After embedding, specimens were sectioned at a thickness of 5 micrometers from the medial side to the cruciate ligament junction. Safranin O/Fast green staining and H&E staining were performed. The severity and progression of OA were assessed using Mankin scores and synovitis scores, which were conducted after Safranin O and H&E staining. For IHC staining, sections were incubated with the following primary antibodies: anti-Col2a1 (1:100, Bioss Antibodies, Woburn, MA, USA; bs-0709R), anti-MMP-13 (1:100, Proteintech, Rosemont, IL, USA; 18165-1-AP), anti-CD68, (1:200, Proteintech; 28058-1-AP) anti-IL6 (1:200, Affinity Biosciences Ltd., Cincinnati, OH, USA; DF6087), anti-TNF-α (1:200, Proteintech; 17590-1-AP). After two rinses, sections were incubated with appropriate secondary antibodies and photographed using an optical microscope VS200 (Olympus, Tokyo, Japan; IX73P1 F).

### Fluorescence imaging

After collecting mouse knee joints treated with MNs loaded with Cy5.5-labeled De, they were immersed in a 4% formaldehyde solution overnight. Subsequently, decalcification was performed in 10% EDTA for five days, followed by cryosectioning. To better observe the specific location of fluorescence distribution, the specimens were sectioned into 20-micrometer slices. Since the samples were inherently fluorescent, no further incubation with antibodies was required. The entire process was carried out in the dark, and finally, a full-spectrum wavelength confocal microscope was used for imaging.

### Magnetic resonance imaging

After 2 months of treatment, magnetic resonance imaging (MRI) scans of canine joints were performed on a 3T MAGNETOM Skyra (Siemens Healthineers, Erlangen, Germany) equipped with a dedicated peripheral knee coil using the double echo steady state (DESS) sequence. The MR images of the canine joint were manually divided into three sub-regions, and the femoral articular cartilage thickness in the central region was quantitatively assessed. Additionally, the synovium was graded using the MOAKS (Modified Osteoarthritis Knee Society) classification from both sagittal and transverse images.

### Real-time polymerase chain reaction (RT-PCR)

Total RNA was extracted from the synovial tissue of SD rats using FreeZol reagent (Invitrogen, Carlsbad, CA, USA) according to the manufacturer's protocol, and cDNA was reverse transcribed using a reverse transcription kit (TaKaRa Bio Inc., Shiga, Japan). TB Green Premix Ex Taq II (TaKaRa Bio) was used for RT-PCR detection. All primer sequences for RT-PCR are listed in Table S1.

### Enzyme-linked immunosorbent assay (ELISA)

The levels of TNF-α, IL6 and MMP13 in the joint fluid and serum were measured using a canine IL-6 ELISA kit (JYM0011Ca), a canine TNF-α ELISA kit (JYM0005Ca), and a canine MMP13 ELISA kit (JYM0018Ca). The levels of IL-1β, IL6, MMP9 and MMP13 in the supernatant of senescent macrophages were measured using a mouse MMP9 ELISA kit (JYM0737Mo), a mouse MMP13 ELISA kit (JYM0559Mo), a mouse IL-6 ELISA kit (JYM0012Mo), and a mouse IL-1β ELISA kit (JYM0531Mo). The levels of creatinine (crea), urea, alanine aminotransferase (ALT) and aspartate aminotransferase (AST) in the serum were measured using a mouse crea ELISA kit (R05602), a mouse urea ELISA kit (R02902), a mouse ALT ELISA kit (R01502) and a mouse AST ELISA kit (R01702), according to the manufacturer's protocol.

### Western blotting

Total protein was harvested from cells or tissues using RIPA lysis buffer (Beyotime Institute of Biotechnology, Jiangsu, China) supplemented with phosphatase and proteinase inhibitors, and the concentration was determined by the BCA assay (Beyotime Institute of Biotechnology). Proteins were subjected to sodium dodecyl sulphate polyacrylamide gel electrophoresis (SDS-PAGE) and transferred onto a 0.45 μm polyvinylidene difluoride membrane. The membrane was then incubated overnight at 4°C with the following primary antibodies: anti-CD86 (1:1,000, Proteintech, 13395-1-AP), TNF-α (1:1,000, Proteintech, 17590-1-AP), ARG1 (1:1,000, Affinity, DF6657), P16 (1:1000, ABclonal, Woburn, MA, USA; A23882), P21 (1:1000, ABclonal, A19094). After washing with TBS containing 0.01% Tween 20 (TBST) three times, horseradish peroxidase-conjugated secondary antibodies were added and incubated for 1 h at room temperature. Signals on the membrane were visualized using enhanced chemiluminescence (ECL).

### Cell culture

RAW264.7 cells (TIB-71™; ATCC, Manassas, Virginia, USA) were cultivated at 37°C and 5% CO_2_ in Dulbecco's Modified Eagle Medium (DMEM BL301A, Biosharp, Hefei, China) with 10% fetal bovine serum, and penicillin-streptomycin (100 U/mL).

Chondrocytes were harvested from articular cartilage of 3-day-old mouse knee joints. Cartilage samples were harvested, cut up into pieces using a sterile scalpel under a dissection microscope and digested in 0.25% trypsin for 0.5 hours. Then the pieces of cartilage were further digested with 900 U/mL collagenase Type I for 2 h. Dissociated cells were centrifuged and resuspended in DMEM containing 10% FBS, penicillin (100 U/ mL), and streptomycin (100μg/mL).

### Induction of senescence using TBHP

Cells were treated with 50 μM TBHP by pipetting the appropriate volume of TBHP (diluted in DMEM) directly into the medium. Cells were treated with 50 μM TBHP for 24 hours, with the medium changed every 24 hours, for a total of three treatments. After the last treatment, culture was continued for another 24 hours before conducting experimental assays.

### Cytochemistry for SA-β-galactosidase

To evaluate the activity of SA-β-galactosidase, the cells were stained as described (Solarbio, Beijing, China; G1580). The percentage of senescent cells was calculated by dividing the number of blue positive cells by the total cell number in a given area. At least 400 cells were counted per group.

### Conditioned medium

RAW264.7 cells were seeded into a 6-well plate and incubated with TBHP or TBHP + De for 24 h. They were then washed three times with PBS and the culture medium was changed to serum free DMEM. After another 24 h, the conditioned medium was collected for further experiments.

### Statistical analysis

Data are represented as the mean values ± standard deviation (SD) and all statistical analyses and mapping were performed using GraphPad Prism 8 software (GraphPad Software Inc., La Jolla, CA, USA). Two groups were compared by *t* test. One-way analysis of variance (ANOVA) and two-way ANOVA were used for multiple comparisons. Values of *P* < 0.05 were considered significant.

## Supplementary Material

Supplementary figures and table.

## Figures and Tables

**Figure 8:**
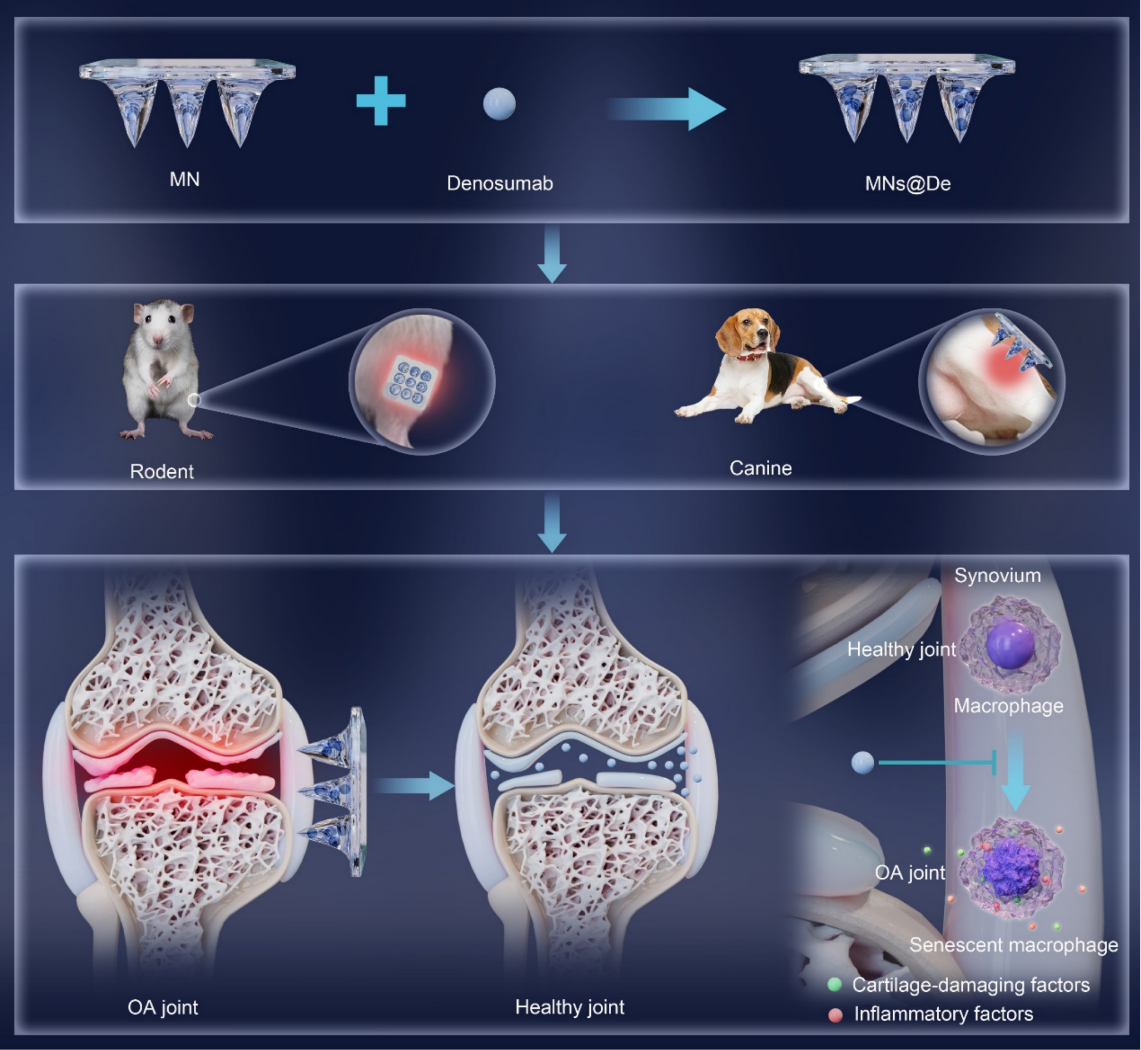
**Schematic illustration of a single application of dissolving microneedles loaded with denosumab (MNs@De) for long-term treatment of osteoarthritis (OA).** The diagram shows the fabrication process of a MNs@De by loading denosumab into microneedles (top). The MNs@De is then applied to the knee joints of rodent and canine models for treatment (middle). Short-term use of MNs@De effectively relieves the progression of OA, protects cartilage from OA-related degradation, and prevents synovitis (bottom left). At the cellular level, senescent macrophages in arthritic synovium are significantly increased, releasing pro-inflammatory and cartilage-damaging factors. Denosumab alleviates macrophage senescence in the synovium, thereby protecting cartilage and suppressing inflammation (bottom right).
