# Supplementary material for: Dissolvable microneedles loaded with denosumab alleviate knee osteoarthritis in rodent and canine models by inhibiting macrophage senescence
Source: Theranostics. 2026 Jan 1;16(1):325–44. doi: 10.7150/thno.116970 (PMC12665124; doi:10.7150/thno.116970)
Supplement: Supplementary file 1 — Supplementary figures and table. [file thnov16p0325s1.pdf]

Supplementary Materials:

Table S1: Real-time PCR primer sequences used in this study

Gene	Forward primer (5' to 3')	Reverse primer (3' to 5')
RAT-TNF-α	CCGAGATGTGGAACTGGCAGAG	CCACGAGCAGGAATGAGAAGAGG
RAT-IL-1β	TCTCACAGCAGCATCTCGACAAG	CCACGGGCAAGACATAGGTAGC
RAT-GM-CSF	GATGCCGCTTGTCAGTGCTAAC	GGTCGTAATGCTGCTGCCTATTG
RAT-GAPDH	ACGGCAAGTTCAACGGCACAG	CGACATACTCAGCACCAGCATCAC
MOU- GAPDH	GGCAAATTCAACGGCACAGTCAAG	TCGCTCCTGGAAGATGGTGATGG
MOU-COL2A1	ACGAGGCAGACAGTACCTTGAG	TGGTTGTTCAGTGACTTGAGTGTAG
MOU-COLX	GATGCCGCTTGTCAGTGCTAAC	GGTCGTAATGCTGCTGCCTATTG
MOU-MMP13	CAGTTGACAGGCTCCGAGAAATG	CACATCAGGCACTCCACATCTTG
MOU-ACAN	TCCGACATAGACACAGGCACTTC	GCTGATGGCAACATTCACCTCTG
MOU-ADAMTS5	GCTCCTCTTGGTGGCTGACTC	GGCGGATGTGGTTCTCAATGC
MOU- IL-6	GGCTAAGGACCAAGACCATCC	GCACTAGGTTTGCCGAGTAGA
MOU-STAT1	GCCTCTCATTGTCACCGAAGAAC	TGGCTGACGTTGGAGATCACCA
MOU- CCL2	GCTACAAGAGGATCACCAGCAG	GTCTGGACCCATTCCTTCTTGG
MOU- CCL24	ATTCCAGAAAACCGAGTGGTTAGC	GCATCCAGTTTTTGTATGTGCCTC
MOU- CCL8	GGGTGCTGAAAAGCTACGAGAG	GGATCTCCATGTACTCACTGACC

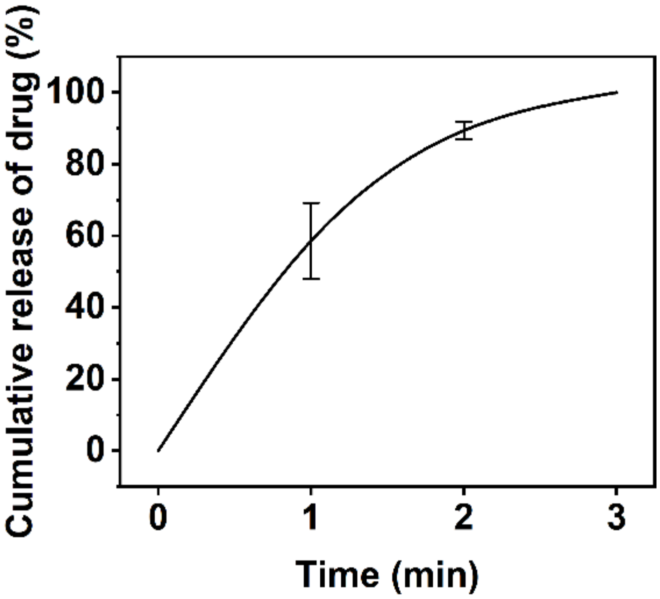

Figure S1: The cumulative release curve of FITC-labeled BSA from PVP MNs following immersion in water.

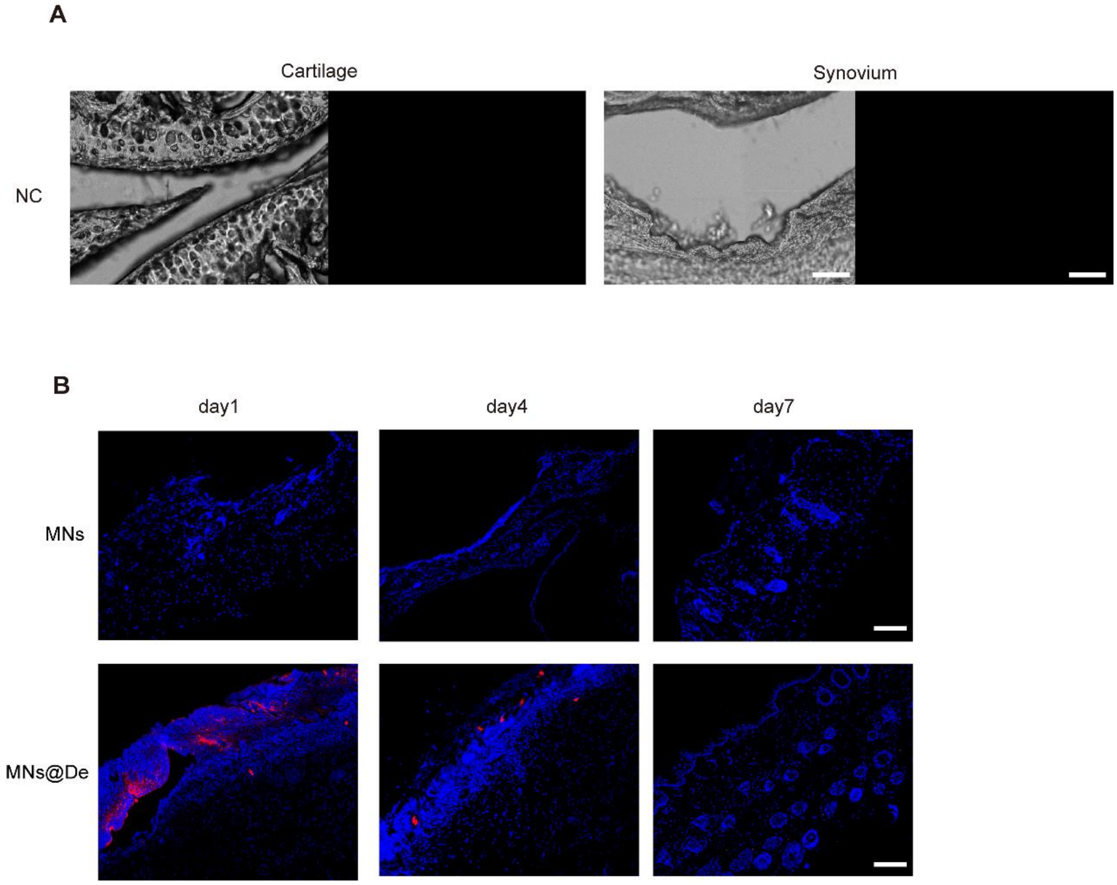

Figure S2: The drug diffusion of MNs@De in the skin surface of the knee joint.

(A) Negative control group for confocal fluorescence imaging. (B) Fluorescence images of the blank microneedle group and the MNs@De group on the skin surface of the knee joint, with blue representing DAPI and red representing Cy5.5-labelled denosumab. Scale bars, 100 μm.

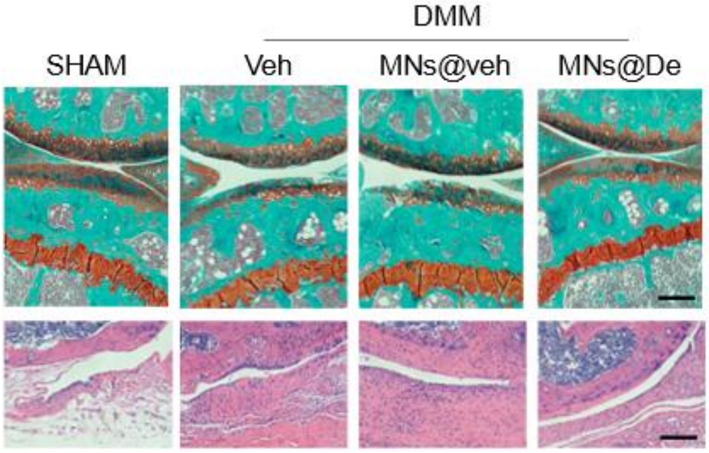

Figure S3: Examples of Safranin O and H&E staining in SHAM, DMM, DMM+MNs@veh, DMM+MNs@De mice. Scar bar,200um.

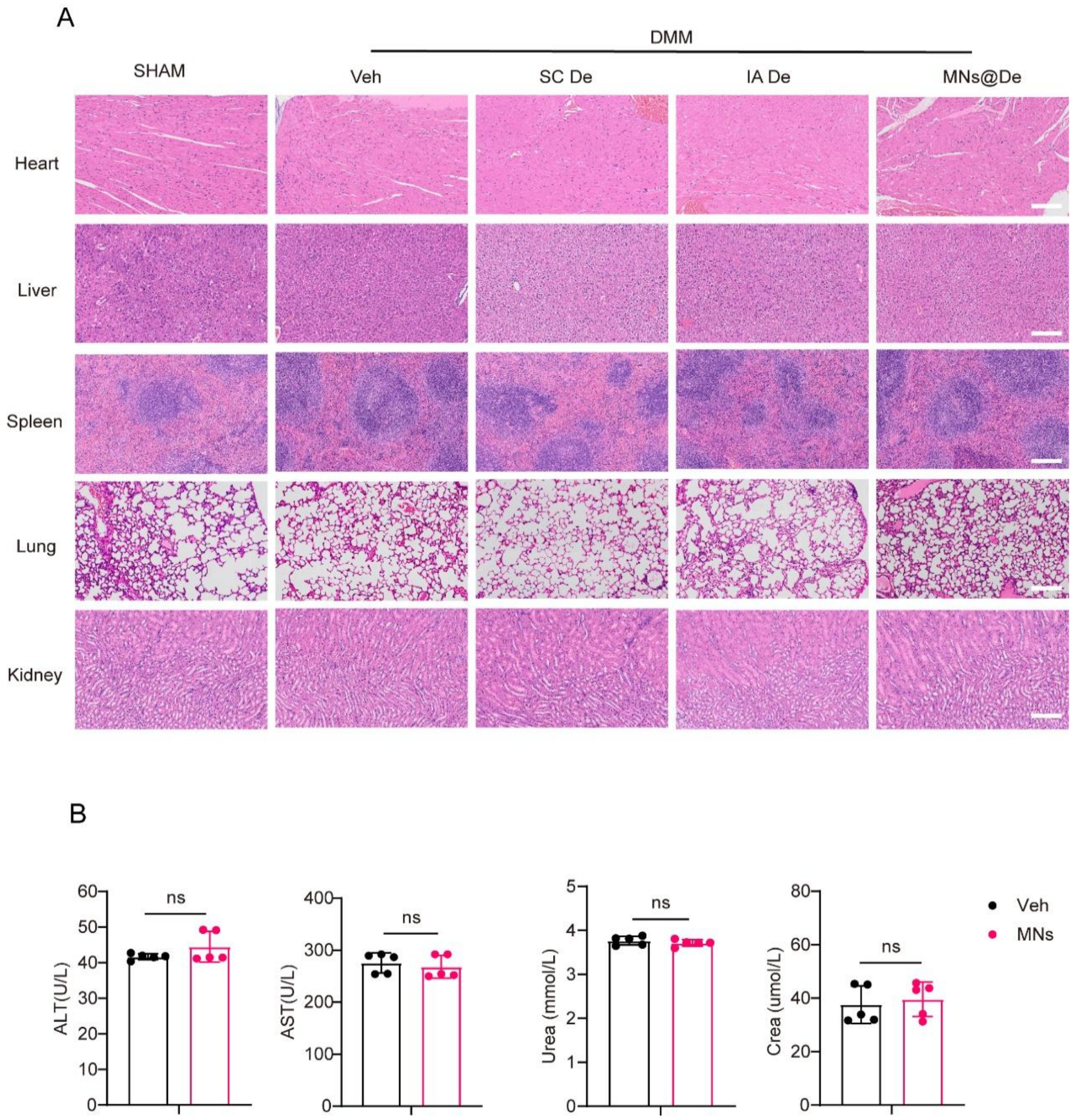

Figure S4: Microneedle toxicity testing on major organs of mice.

(A) H&E staining of organs(heart, liver, spleen, lung, kidney) in sham,dmm,SC De, IA De and MNs@De mice. Scar bar, 100um. (B) Detection of liver and kidney function indicators (ALT, AST, Urea, Crea) in Veh and MNs mice. (n = 5 per group).

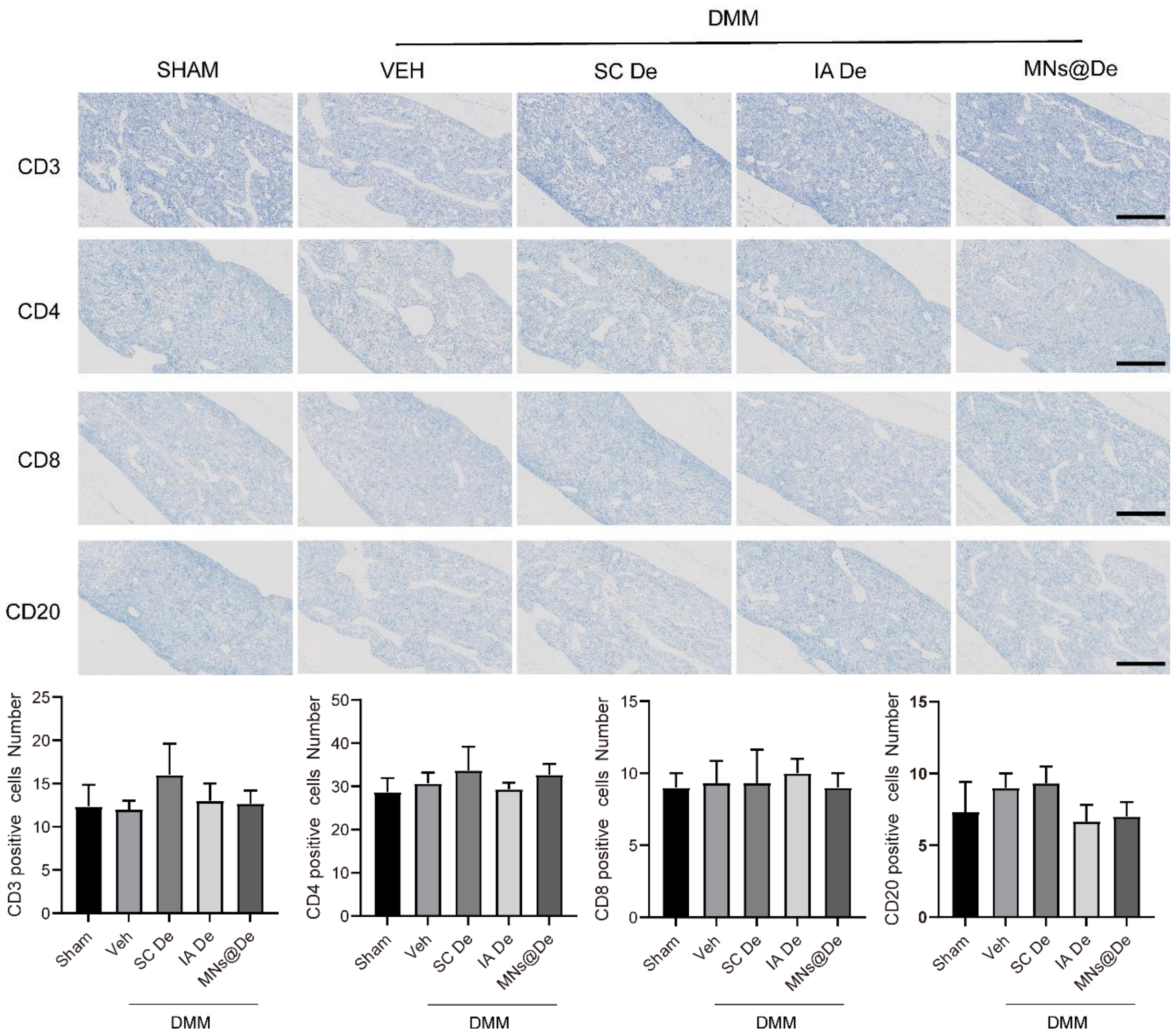

Figure S5: Immunohistochemical staining images and quantification for CD3, CD4, CD8 and CD20 in sham, DMM, SC De, IA De and MNs@De mice bone marrow; scale bar = 200 μm.

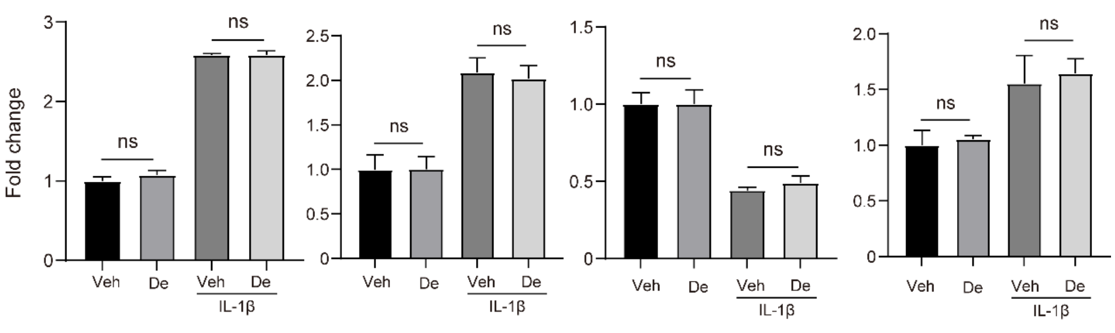

Figure S6: RT-qPCR was performed to assess the mRNA levels of MMP13,

ADAMTS5,COLII and COLX in chondrocyte from the Veh, De, IL-1β+veh, and IL-1β+De groups ($n = 5$ per group). Statistical analyses were conducted using one-way ANOVA followed by the by Dunnett multiple comparisons test. Error bars are mean ± SD, * $P < 0.05$, **$P < 0.01$, ***$P < 0.001$, ****$P < 0.0001$.

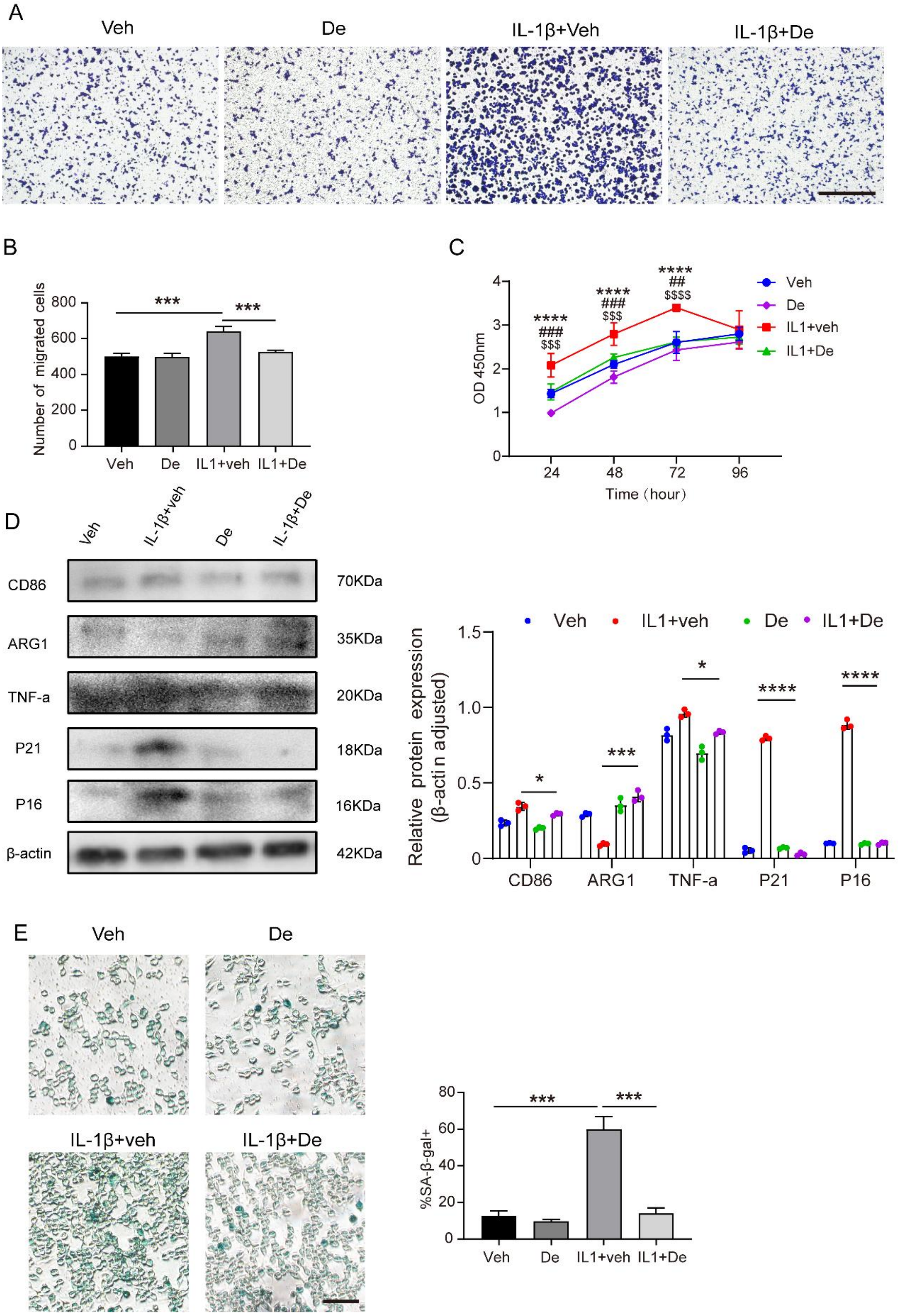

Figure S7: Denosumab reduces macrophage migration and proliferation, and polarizes them towards the M2 phenotype.

(A) Representative transwell migration assay of macrophages.

Macrophages were seeded on the upper chamber at 2,000 cells/mL, then IL-1β or denosumab was added to the lower chamber. After 12 hours, migrated cells on the lower surface of the Transwell™ membrane were stained with crystal violet. Scar bar,100um.(B) Semi-quantitative analysis of macrophage migration numbers. (n = 3 per group). (C) The CCK-8 assay measures absorbance at 450 nm to reflect the proliferation of macrophages.*,Veh vs IL-1β;#,IL-1β VS IL-1β+De;$,IL-1β+De vs De. (n = 5 per group). (D) Western blot analysis of CD86, TNF-a, ARG1,P16,P21in vehicle, IL-1β, IL-1β + De and De treated Macrophages(n = 3 per group).(E) SA-β-gal staining of macrophages induced to inflammation by IL-1β and semi-quantitative analysis,scar bar,200um.(n = 3 per group). Statistical analyses were conducted using one-way ANOVA followed by the by Dunnett multiple comparisons test.Error bars represent mean ± SD, * P < 0.05, **/##P < 0.01, ***/###/$$$$P$<0.001,****/$$$$$P$ < 0.0001.

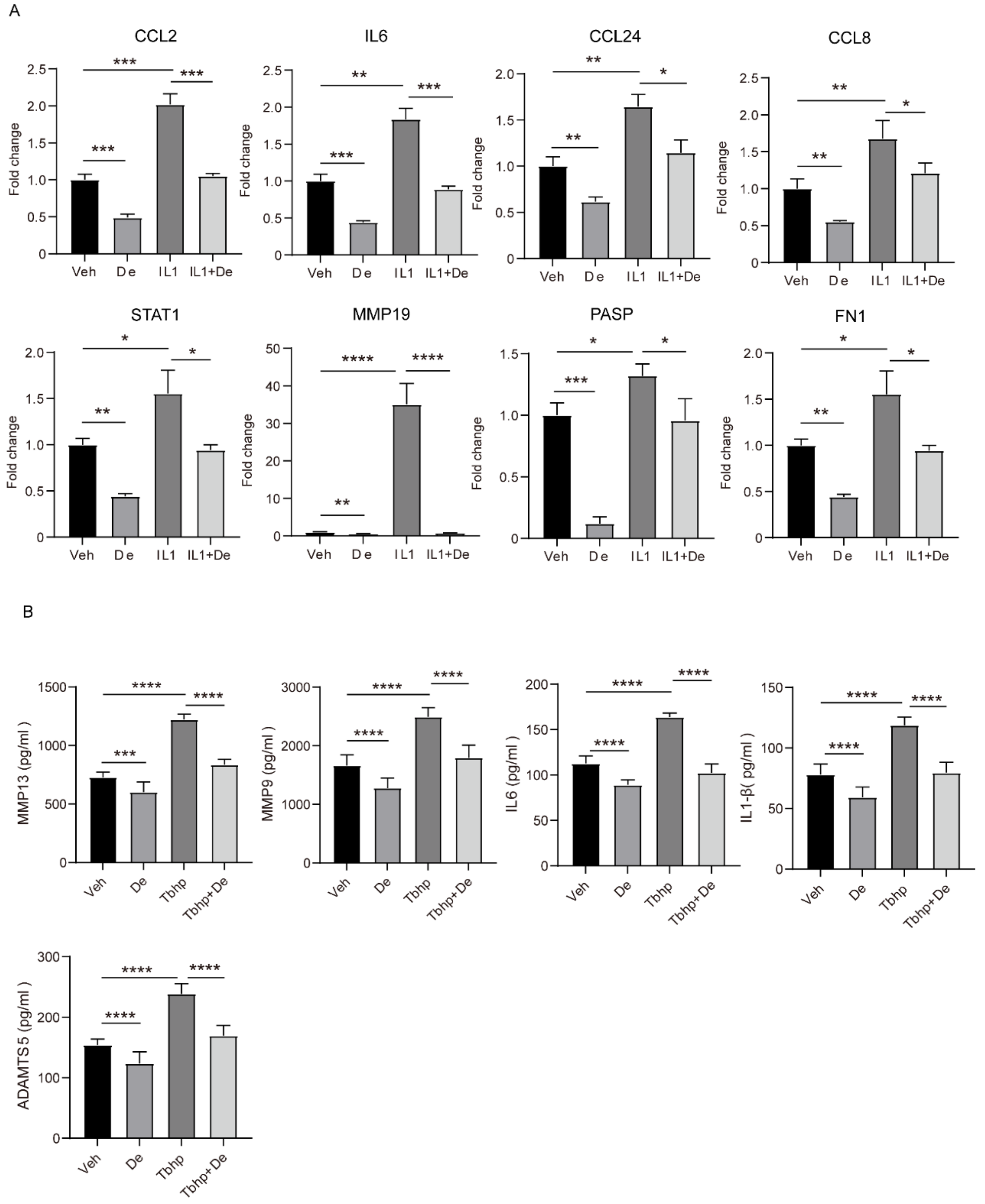

Figure S8: (A) RT-qPCR was performed to assess the mRNA levels of CCL2, CCL8, CCL24, IL6, STAT1, MMP19, PASP, and FN1 in macrophages from the Veh, De, IL-1β+veh, and IL-1β+De groups (n = 5 per group). (B) The concentrations of MMP13, MMP9, IL-6, IL-1β, and ADAMTS5 in macrophages from the Veh, De, Tbhp, and Tbhp+De groups were assessed by enzyme-linked immunosorbent assay (n = 8 per group).

Statistical analyses were conducted using one-way ANOVA followed by the by Dunnett multiple comparisons test. Error bars are mean ± SD, * $P < 0.05$, ** $P < 0.01$, *** $P < 0.001$, **** $P < 0.0001$.

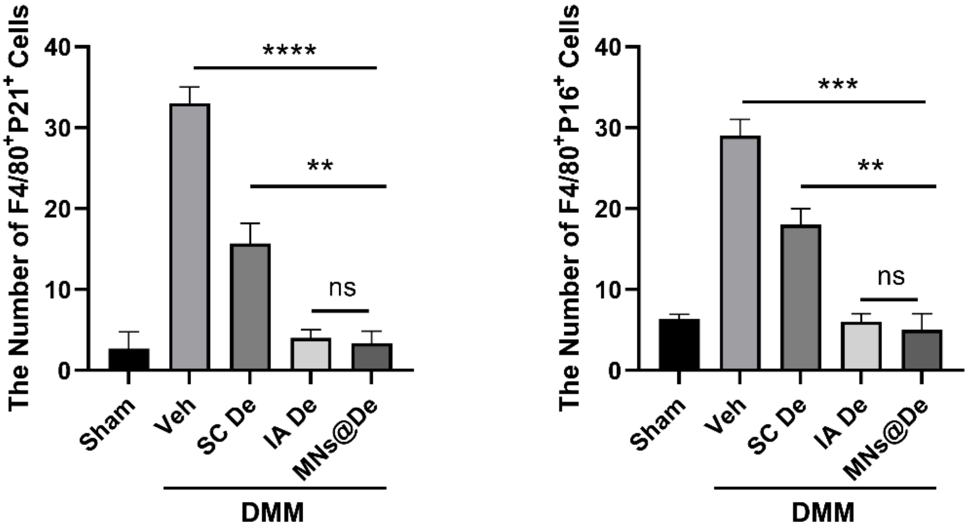

Figure S9: quantification of P16/P21-positive cells in synovial tissues from mice in the Sham, DMM+Veh, DMM+SC De, DMM+IA De, and DMM+MNs@De groups.(n=5) Statistical analyses were conducted using one-way ANOVA followed by the by Dunnett multiple comparisons test. Error bars are mean ± SD, * $P < 0.05$, ** $P < 0.01$, *** $P < 0.001$, **** $P < 0.0001$.

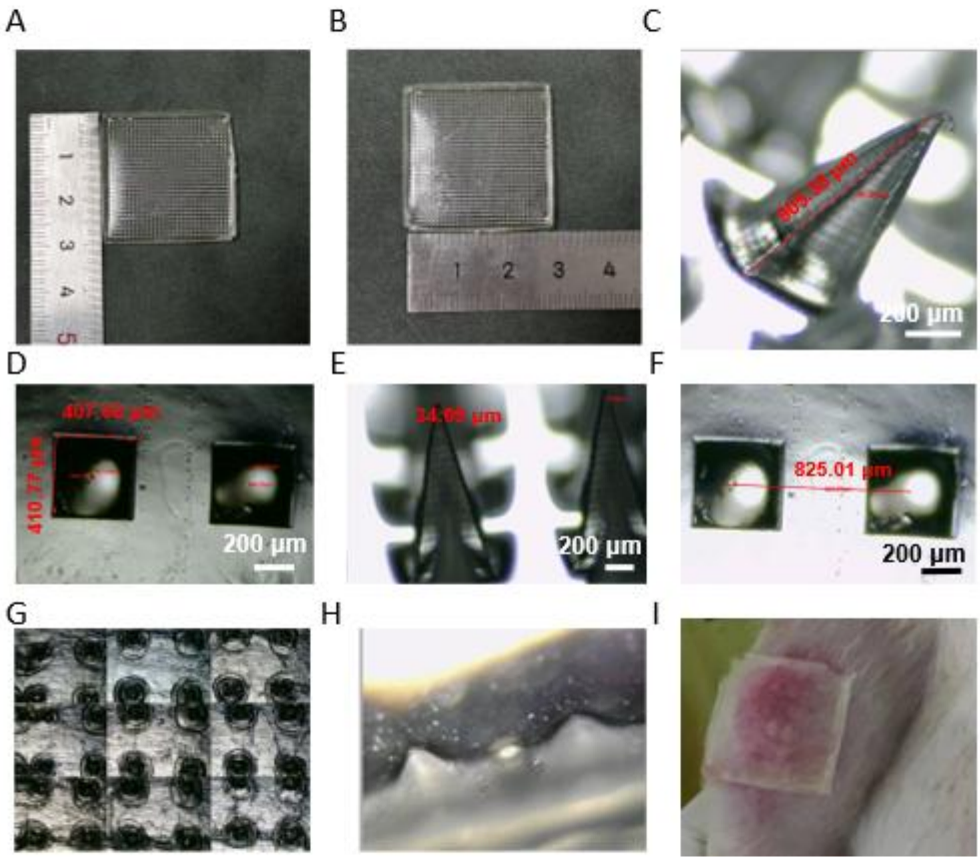

Figure S10: Characterization of MN patch for large animal (dog) Applications.

(A ,B) Dimensional analysis of MN patch length and width (3 cm × 3 cm). (C) Morphological characterization and height measurement of microneedle tips (805-905 μm). (D) Quantitative assessment of base dimensions (404-435 μm) and (E) Tip apex width for individual MN measurement (30-44 μm). (F) Inter-needle spacing analysis between adjacent MN (819-844 μm). (G) Penetration capability evaluation through the five-layer parafilm M barriers of MN. (H) In vitro dissolution of MN patch in aqueous medium. (I) Photograph of MN patch application on the dog joint anatomy.

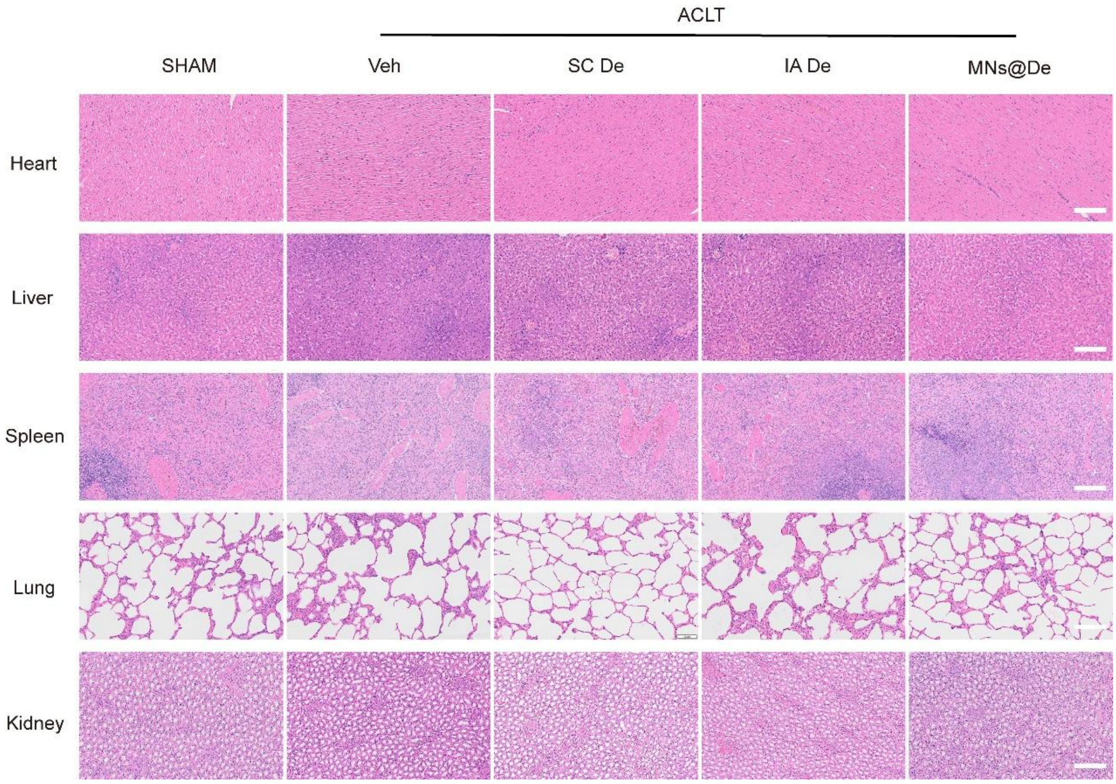

Figure S11: Microneedle toxicity testing on major organs of beagles.

H&E staining of organs (heart, liver, spleen, lung, kidney) in SHAM, ACLT, SC De, IA De and MNs@De beagles. Scar bar, 100um
